# The p53 DNA-Binding (Core) Domain can form a prion that seeds prion formation in full-length p53

**DOI:** 10.15698/mic2026.10.887

**Published:** 2026-10-02

**Authors:** Mathilde Kadouch, Christelle Marchal, Sei-Kyoung Park, Sangeun Park, Sabine Castano, Susan W. Liebman, Christophe Cullin

**Affiliations:** 1 Univ. Bordeaux, CNRS, Bordeaux INP, CBMN, UMR 5248, F-33600 Pessac, France; 2 Department of Pharmacology, University of Nevada, Reno, United States of America

**Keywords:** yeast, p53, prion, prionization frequency

## Abstract

The p53 tumor suppressor is frequently inactivated in human cancers, often through mutations in its core-domain. Here, we developed a yeast reporter system in *S. cerevisiae* to investigate whether the p53 core-domain can function as a prion-forming domain. By using a chimeric protein (CD-FDp) combining the p53 **C**ore **D**omain and the **F**unctional **D**omain of the yeast prion protein Ure2p, we demonstrate that inactive CD-FDp forms heritable, self-propagating aggregates. These aggregates exhibit canonical prion properties, including dominance, non-Mendelian segregation, and cytoduction-mediated transmission. CD-FDp prionization is independent of canonical chaperones (Hsp104, Hsp70, Hsp90), distinguishing it from classical yeast prions. Prionized CD-FDp enhances the formation of amyloid-like foci in full-length p53-EYFP, as evidenced by Thioflavin T staining, and can transmit its prion state to full-length p53 via cytoplasmic transfer. This indicates that CD-FDp propagons can induce structural conversion of p53, supporting an autocatalytic aggregation model. Using a Luria-Delbrück fluctuation assay, we quantified the prionization propensity of wild-type and cancer-associated p53 core-domain mutants. While most mutants (e.g., R273H, R282W) showed inactivation frequencies similar to wild-type, the R175H mutant exhibited an ∼ 18-fold increase in prionization frequency, which correlates with its known aggregation propensity in human cancers. This yeast system thus provides a quantitative platform to assess the prion-like behavior of p53 mutants and screen for inhibitors of p53 inactivation. Our findings establish p53 core-domain as a novel, structurally distinct prion-forming domain and offer a powerful experimental framework for studying p53 inactivation mechanisms, with potential implications for cancer therapeutics.

## INTRODUCTION

The transcriptional activator p53 (gene ID: 7157, NIH) mediates a variety of cellular processes, including cell-cycle arrest, DNA repair, and apoptosis. At the structural level, it contains several domains: an N-terminal domain (residues 1–93) that is intrinsically disordered and functions as a transcriptional activator, a central core-domain (102–292) that is highly structured and responsible for DNA binding, and a C-terminal domain (292–393) responsible for nucleocytoplasmic shuttling and tetramerization. Within the core-domain, an aggregation-nucleating segment spanning residues 251–257 was identified as important to destabilize and aggregate p53 1. The sequence (250–257), named P8, was subsequently shown to form amyloid fibrils *in vitro* that can seed aggregation of the core-domain 2. Notably, P8 fibrils, but not monomeric P8, seed the aggregation of endogenous soluble p53 in cell culture 2.

P53 inactivation is a key event in many pathogenic situations. It has been largely documented in association with cancer, but also during viral infection 3. P53 mutations are found in more than 50% of human cancers 4, leading to a loss of function and/or aggregation of the protein 5. Evidence linking p53 aggregation to cancer has accumulated over several decades. As early as 1991, Kraiss et al. 6 demonstrated a correlation between high-molecular-weight p53 oligomers and cellular transformation. Amyloid aggregates co-localizing with p53 have since been reported in human breast cancer tissues 7, as well as human lung, urothelial, mouse colon carcinoma, and rat hepatocarcinoma 8. *In vitro*, the p53 core-domain can form amyloid assemblies 9, and these aggregates can induce aggregation of wild-type (WT) p53 in cells upon transfection, leading to the loss of p53 function 8. Strikingly, co-culture experiments showed that aggregates formed in one cell can be transmitted to naïve cells 8, a mechanism reminiscent of prion-like propagation.

Like the original infectious prion protein PrP, a prion-like protein must be capable of adopting at least two distinct conformational states. In yeast, the alternative conformation is identified as a functional change leading to a new phenotype. While many proteins can be destabilized in a cellular context, and may therefore exist in multiple folding states, this alone does not confer prion-like behavior. The hallmark of prion-like proteins is the ability of the alternative conformation to recruit natively folded copies of the same protein in a self-templating, exponential mechanism driven by fragmentation and elongation cycles. As a result, in yeast, the alternative state is transmitted from mother to daughter cell. In addition, yeast prions are characterized by their genetic properties: the phenotype associated to the alternative conformation is dominant, exhibits non-Mendelian segregation in genetic crosses and can be transmitted by cytoduction. The concept of “prion strains”, called “prion variants” in yeast, derives from the pathogenic agent PrP 10 and represent distinct misfolded PrP conformations and assembly 11. In yeast, this phenomenon has been demonstrated in prionized-Sup35p ([*PSI* ${}^{+}$ ]) strains. *In vitro*, Sup35p can form distinct, self-perpetuating structured amyloid fibers 12. *In vivo*, among others, are found “weak” and “strong” [*PSI* ${}^{+}$ ] variants, leading to weak or strong ability to suppress nonsense mutations respectively 13.

We previously demonstrated in *S. cerevisiae* that full-length p53 can form a self-propagating aggregate with properties characteristic of a yeast prion 14. Overexpression of p53 in yeast induces the formation of a heritable prion state. A characterized resulting prion strain exhibited reduced p53 activity, and in 30–50% of cells, p53-EYFP formed cytoplasmic dots that stained with Thioflavin T (ThT). In this prion strain, p53 is predominantly found in the pellet fraction following ultracentrifugation of crude cell extracts. Diffuse p53-EYFP forms cytoplasmic dots after cytoduction with the prion strain or after transfection with a cellular lysate from a prion strain or *in vitro*-made P8 fibers. Finally, the stable production of p53-EYFP dots is sustained by the continued presence of p53 and is lost upon its transient absence.

Notably, unlike the canonical yeast prions [*URE3*] and [*PSI* ${}^{+}$ ], p53 lacks an asparagine (N)- and glutamine (Q)-rich prion domain, and its propagation is independent of the chaperone Hsp104. These characteristics are shared with a recently described class of prion-like elements in yeast driven by non-Q/N-rich intrinsically disordered sequences 15. Together, these findings established p53 as capable of prion-like behavior, with significant implications for cancer biology.

Using a complementary yeast-based system, we show here that the core-domain of p53 alone is sufficient to drive prionization. When fused to the functional domain of Ure2p, the core-domain gives rise to a chimeric prion with a strong, p53-function-independent phenotype that enables direct quantification of prion formation frequency and isolation of candidate prion variants — both of which were unachievable with our prior assay 14. Using this system, we demonstrate that the prion conformation of the core-domain can seed prion formation in full-length p53, linking the new findings directly to our earlier work. We also showed that the well-characterized cancer mutation R175H dramatically increases the propensity of the core-domain to switch into a prion state. Finally, we showed that non-prion strains exhibit resistance to radicicol, providing a selectable phenotype that could be exploited in screens for genes or compounds that modulate p53 prionization. Together, these results not only corroborate the p53 prion hypothesis through a convergent experimental approach, but provide new tools and a quantitative framework for identifying therapeutic strategies targeting p53 aggregation in cancer.

## RESULTS

### A new yeast reporter for the ability of the p53 core-domain to form a prion

The reporter system we developed to monitor p53 core-domain prion formation is independent of p53 function. Rather, it depends on the activity of the functional domain of a *bona fide* yeast prion protein, Ure2p. Like all canonical yeast prions, Ure2p is composed of a prion and a functional domain. The prion domain controls all Ure2p’s prion properties and is in the N-terminal region. The C-terminal regions is a GST-like functional domain (FD) 16 which starts at methionine 94 and continues to the end of the protein 17. Here, the Ure2p prion domain was replaced by the core-domain (CD) of p53 (Figure 1**A**) that we wanted to test as a possible prion domain. The resulting chimeric ORF (CD-FDp) encoding the p53 **C**ore-**D**omain fused to the **F**unctional Ure2 **D**omain was placed under the control of the *GPD* promoter in a plasmid with a *TRP1* marker to form pGPDW-CD-FD, which was transformed into the MK1 reporter strain. MK1 carries a deletion of *URE2* and has *ADE2* under the control of the *DAL5* promoter. As the allantoate permease gene *DAL5* is activated by Gln3p in *ure2* Δ strains, MK1 is Ade ${}^{+}$ and white. Transformants with pGPDW-CD-FD become Ade ${}^{-}$ and pink, indicating that CD-FDp is functional and its Ure2FDp sequesters the transcription activator Gln3p away from the *DAL5* promoter (Figure 1**A, B**). The positive control (pGPDW-URE2 transformants expressing full-length Ure2p) is Ade ${}^{-}$ and red. The difference in color is likely due to a slightly less efficient sequestration of Gln3p by CD-FDp compared to Ure2p.

In an effort to isolate prion forms of the chimeric protein, we selected for pGPDW-CD-FD transformants that became Ade ${}^{+}$ on plasmid-selective medium lacking adenine. We reasoned that in these clones, pGPDW-CD-FD may no longer be able to produce much functional CD-FDp (CD-FDp ${}^{F}$ ) because the protein forms a prion instead. We tested the presence of CD-FDp in seven Ade ${}^{+}$ pGPDW-CD-FD transformants by western blot. Three expressed it at a level comparable to the Ade ${}^{-}$ transformant (data not shown). We retested one of these Ade ${}^{+}$ transformants, confirming that genetic events could not have inactivated the chimera, because the total expression signal of CD-FDp in both Ade ${}^{-}$ and Ade ${}^{+}$ strains (i.e., strains in which CD-FDp is active or not, respectively) were comparable (Figure 1**C**, lanes T). The CD-FDp in this Ade ${}^{+}$ clone is thus inactivated by a post-translational mechanism which is maintained during mitosis. Furthermore, in the Ade ${}^{-}$ strain, in which CD-FDp is active, the protein is found mainly in the supernatant (the soluble fraction), whereas it is also found in the pellet fraction when in its inactive form in the Ade ${}^{+}$ clone. This result is consistent with an inactivation by aggregation and is compatible with prionization leading to inactive CD-FDp.

**Figure 1:**
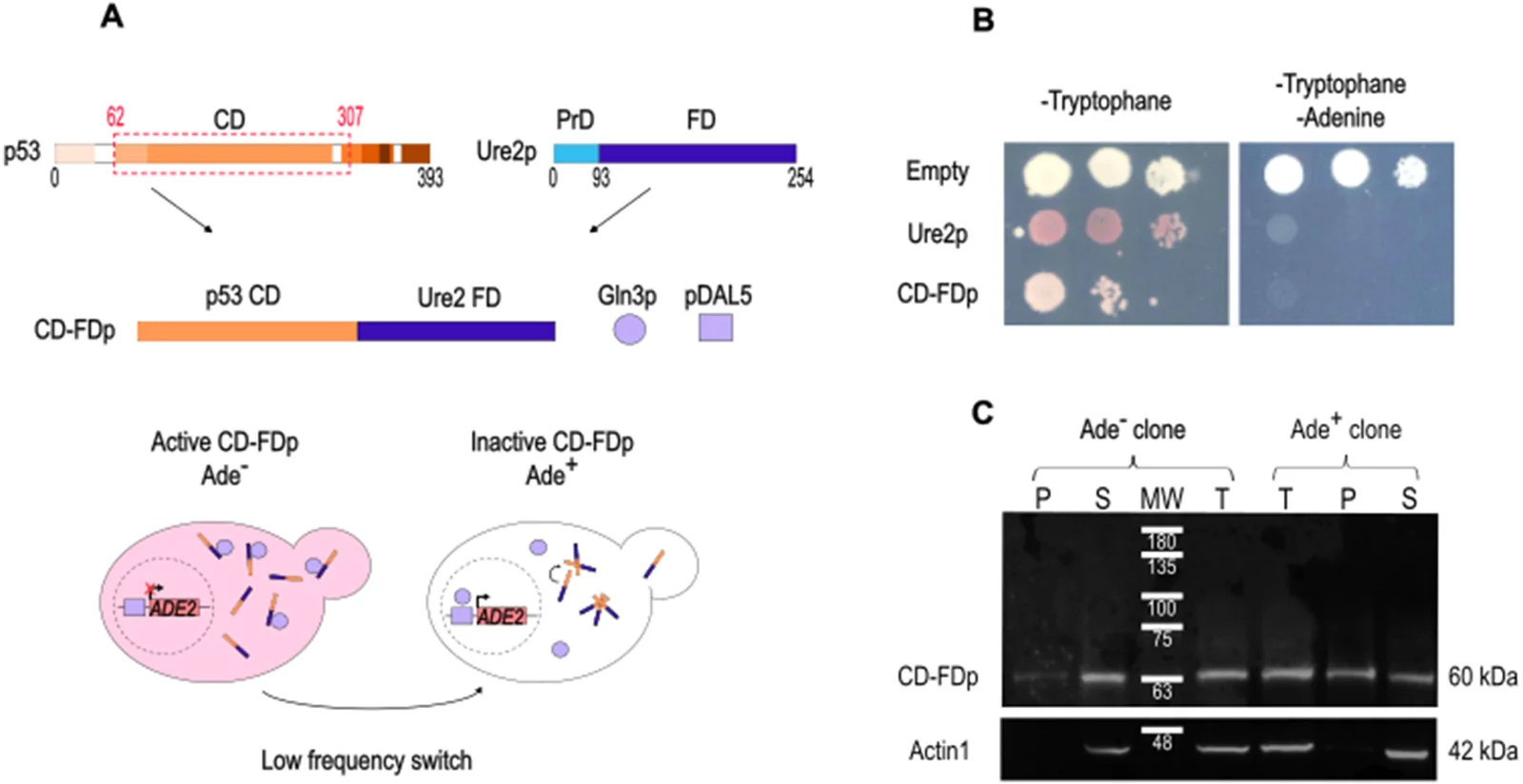
CD-FDp can form two stable conformational states. **(A) Description of the reporter system.** The chimeric protein CD-FDp is composed of a fusion between p53 and Ure2p, in which the N-terminal prion domain of Ure2p is replaced by the core domain of p53. To monitor Ure2p activity, the *ADE2* coding sequence is placed under the control of the *DAL5* promoter at the *DAL5* locus. The nucleus is depicted as a punctate oval. In its active form, CD-FDp sequesters the transcription activator Gln3p, repressing the *DAL5* promoter and preventing *ADE2* expression; as a result, cells are Ade ${}^{-}$ (represented in pink). When CD-FDp is inactivated, Gln3p is released, translocated into the nucleus, and activates *DAL5* transcription, leading to Ade2p production and an Ade ${}^{+}$ phenotype (represented in white). **(B) CD-FDp is functional**. After transformation of MK1 (*ure2* Δ ) with an empty vector (pGPD-ccdB-EGFP) or a plasmid coding for either Ure2p (pGPDW-URE2) or CD-FDp (pGPDW-CD-FD), a pool of transformant was streaked on plasmid selective medium. Tenfold dilutions of the patched cells were spotted onto plasmid-selective plates (without tryptophane) complemented or not with adenine. The cells were incubated at 30 ${}^{\circ }$ C for 3 days. **(C) Inactive CD-FDp is aggregation-prone**. Ade ${}^{-}$ and Ade ${}^{+}$ clones were grown in semi-complete plasmid selective medium and native total protein extracts were prepared as described in the methods section. Following ultracentrifugation at 100,000 × g, equal volumes of total (T), pellet (P), and supernatant (S) fractions were then separated by SDS-PAGE, transferred to a nitrocellulose membrane, and probed for CD-FDp using DO-12 anti-p53 antibodies. After stripping, the membranes were reprobed with anti-actin antibodies as a loading control. MW: Molecular weight. At the right side of the blot are indicated theoretical molecular weights of CD-FDp and actin.

### Inactivated CD-FDp (CD-FDp ${}^{I}$ ) can form a non-Mendelian prion-like element

In *S. cerevisiae,* prions were first identified as “non-classical” genetic elements: the phenotype is dominant and does not follow Mendelian segregation laws 18, 19. Performing such genetic analyses using plasmid-based expression is complicated, due to variable plasmid copy number and inconsistent meiotic segregation. Thus, to identify and perform genetic analyses of potential CD-FDp prions, we integrated the transcriptional cassette built in pGPDW-CD-FD into the MK1 strain at the *LYS2* locus. The resulting *MAT***a** CD-FD strain is Ade ${}^{-}$ , as is MK1 transformed with pGPDW-CD-FD. As a control, we integrated the transcriptional cassette of pGPDW-URE2 to get the GPD-URE2 strain. Both strains are Ade ${}^{-}$ and become Ade ${}^{+}$ on proline medium (Figure 2**A**), which lacks rich nitrogen sources, thereby inhibiting the Ure2p/Gln3p interaction and leading to the activation of genes involved in poor nitrogen source metabolism (such as *DAL5*) 20.

To identify prion candidates, we next selected for Ade ${}^{+}$ clones on synthetic medium lacking adenine, in a homozygous diploid CD-FD strain made from isogenic parents (see Materials and Methods). Seven out of eight Ade ${}^{+}$ clones were shown to still express the chimeric CD-FD protein by western blot (data not shown). After meiosis of three of these candidates, we obtained two patterns of segregation on complete medium. Segregants from one diploid exhibited a classical 2:2 Mendelian segregation of white to red (Figure 2**B**, left) suggesting that the Ade ${}^{+}$ phenotype was due to a dominant genetic mutation.

The other two diploids violated Mendel’s first law of monogenic segregation. For one of these diploids, 14 complete tetrads are displayed (Figure 2**B**, right). Among them, two tetrads displayed a 2:2 (two white spores, two red spores) segregation, eleven tetrads displayed a 3:1 segregation and one displayed a 4:0 segregation. The white and red spores were spotted and confirmed to be Ade ${}^{+}$ and Ade ${}^{-}$ respectively, and the complete tetrads were confirmed to contain two (*MAT***a**) and two (*MAT* α ) haploids. This Ade ${}^{+}$ non-Mendelian segregation pattern is a hallmark of yeast prions.

From two 3:1 tetrads of the above described segregants we chose four potential CD-FD prion haploids. From the first tetrad, the two haploids selected were still white after 5 days on the dissection plates (strains *MAT***a** 1871 and *MAT* α 1870, later referred as prion strain 1, carrying prion variant 1). From the second tetrad, the haploids selected exhibited light pink color after 5 days (strains *MAT***a** 1868 and *MAT* α 1869, later referred as prion strain 2, carrying prion variant 2) (see strain details in Material and Methods). Interestingly, in a spotting assay, the pink clones grew slower than the white ones on medium lacking adenine (Figure S1). Additionally, on the most diluted drops, the appearance of white, pale pink, and dark pink subclones is observed for prion strain 2 (Figure S1). To maintain the pink color, we returned to the glycerol stock every few weeks. CD-FDp was expressed at comparable levels in these strains (supplementary Figure S2). This finding is consistent with the existence of different prion variants in the diploid progenitors of the white and pink prion haploids in which the level of inactivation of CD-FDp is different.

Next, we tested the dominance of the Ade ${}^{+}$ phenotype by crossing the *MAT* α prion strains (prion variant 1 1870 and prion variant 2 1869) with the isogenic *MAT***a** Ade ${}^{-}$ parental strain. Diploids formed by either prion strain mated with the non-prion CD-FD strain became Ade ${}^{+}$ (Figure 2**C**, top). This shows the dominance of Ade ${}^{+}$ phenotype and corresponds to a homotypic inactivation of CD-FDp, which is consistent with classic prion properties. As a control, mating two CD-FD Ade ${}^{-}$ haploids led only to Ade ${}^{-}$ diploid cells.

### The prion form of the p53 core-domain specifically inactivates Ure2p activity in the CD-FDp fusion, but not in Ure2p alone

For the “canonical” yeast prions [*PSI* ${}^{+}$ ] and [*URE3*], the prionization mechanism is highly specific to the prion domain. In other words, aggregated Sup35 will not lead to the aggregation of Ure2p and *vice versa*. It was therefore of interest to test the capacity for Ure2p to be recruited and inactivated by prionized CD-FDp ${}^{I}$ . The prion CD-FD strains mated with the Ade ${}^{-}$ GPD-URE2 strain led to Ade ${}^{-}$ diploids (Figure 2**C**, bottom). This result shows the absence of an efficient heterotypic interaction of CD-FDp with Ure2p. This is consistent with previous findings on yeast prions 21, as the Ure2p prion domain differs from the CD sequence.

To test whether Ure2p expression interferes with the prion form of CD-FDp, we transformed the CD-FD prion strain (1870) with pGPDW-URE2. As a control, we used an empty vector (pGal-ccdB-eGFP) and pGPDW-CD-FD. Pools of transformants were assessed for adenine auxotrophy (Figure 2**D**, top). Empty vector and pGPDW-CD-FD transformants remained Ade ${}^{+}$ while pGPDW-URE2 transformants became Ade ${}^{-}$ , echoing results from mating. Next, we cured pGPDW*-*URE2 transformants of the plasmid by streaking them three times on complete medium. Three independent clones were assayed: they all lost the plasmid and reverted to Ade ${}^{+}$ (Figure 2**D**, bottom). This shows that the reporter was still functional in pGPDW-URE2 transformants and that CD-FDp ${}^{I}$ is maintained independently of Ure2p expression.

**Figure 2:**
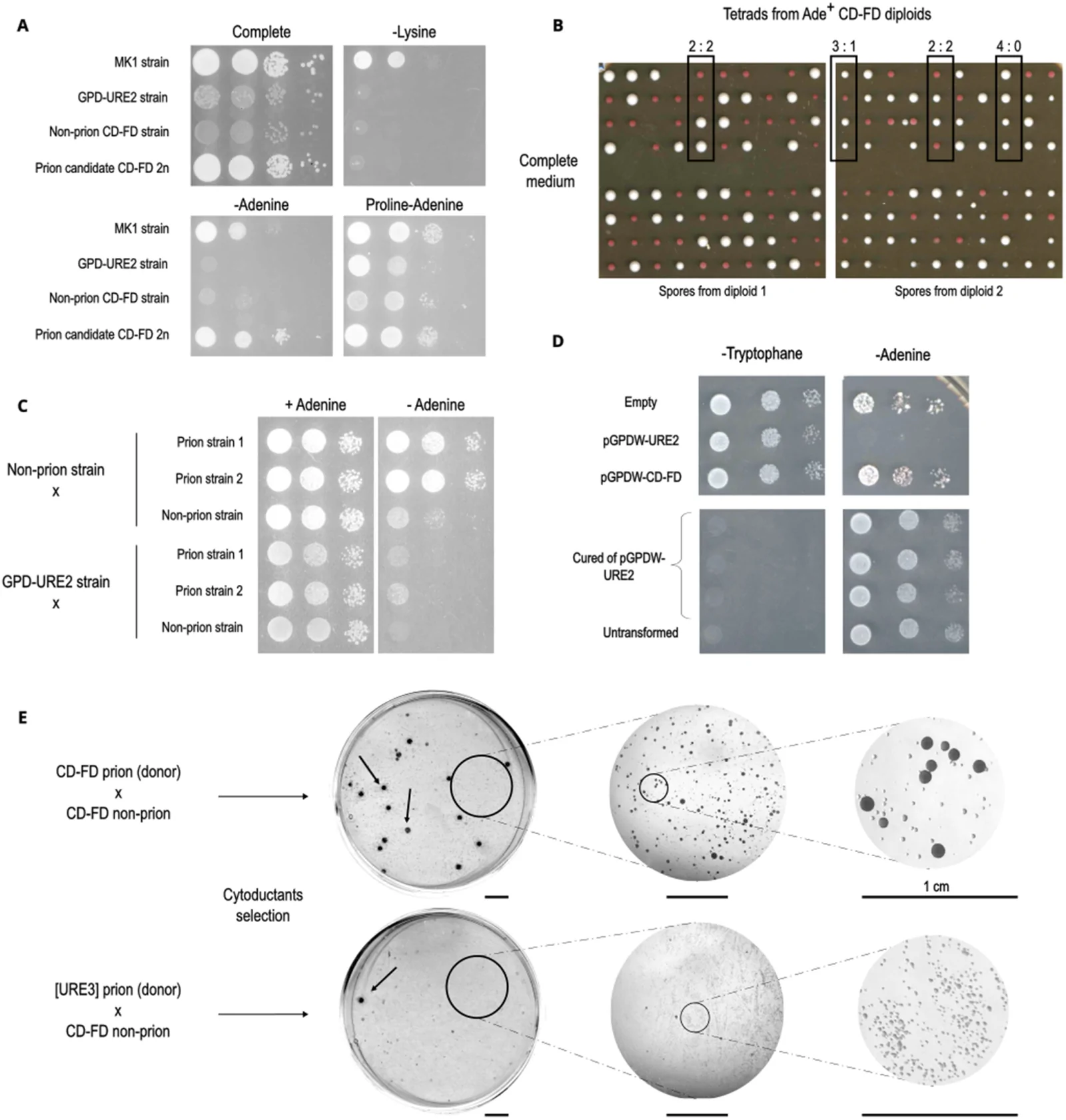
CD-FDp^I^ exhibits genetic properties of yeast prions. **(A) Establishment of integrated strains.** The MK1 *ure2* Δ strain was transformed with a Cas9-encoding vector, then by *LYS2*-targetting gRNA plasmids, as well as with either pGPD-URE2 or pGPD-CD-FD PCR products leading to GPD-URE2 and CD-FD strains respectively. From the diploid Ade ${}^{-}$ CD-FD strain, Ade ${}^{+}$ clones were selected. The MK1 strain before integration, the CD-FD non-prion and GPD-URE2 strain as well as one Ade ${}^{+}$ diploid prion candidate were tested for their auxotrophy. Tenfold dilutions of the cells were spotted on complete medium, semi-complete medium lacking adenine, synthetic dextrose medium lacking lysine and synthetic proline medium lacking adenine. **(B) Non-mendelian segregation of candidate prion strain** Among seven Ade ${}^{+}$ diploids expressing CD-FDp, three were sporulated and their asci were dissected. The left panel illustrates a typical 2:2 segregation pattern (boxed tetrad) while the right panel shows non-Mendelian segregation in asci (boxed tetrads) produced by another diploid (two out of the three dissected diploids exhibit this atypical segregation pattern). Subsequent testing revealed that white colonies were Ade ${}^{+}$ , whereas red colonies were Ade ${}^{-}$ (data not shown). **(C) CD-FDp prion phenotype is dominant.***MAT* α CD-FD prion strain 1 (1870), 2 (1869) and non-prion (1844) strains were transformed with an empty vector with a *TRP1* marker. *MAT***a** CD-FD or GPD-URE2 non-prion strains were transformed with an empty vector with an *HIS3* marker. CD-FD prion and non-prion strains are either mated with CD-FD non-prion (top) or GPD-URE2 (bottom) strains. After 6 h of mating, diploids were selected on semi-complete medium lacking histidine and tryptophane. Tenfold dilutions of pools of diploids were then spotted on semi-complete medium lacking histidine and tryptophane and complemented or not with adenine. **(D) The CD-FDp prion phenotype propagates independently of Ure2p.** The CD-FD prion strain 1 which is Ade ${}^{+}$ (1870) was transformed with an empty vector carrying the *TRP1* selectable marker, the pGPDW-URE2 vector allowing constitutive expression of Ure2p or the pGPDW-CD-FD vector allowing constitutive expression of CD-FDp. Transformants were selected on semi-complete medium lacking tryptophane. Tenfold dilutions of pools of transformants were spotted on semi-complete medium lacking tryptophane or adenine (top). pGPDW-URE2 transformants were then streaked three times on complete medium to cure them of the *TRP1* plasmid. Tenfold dilutions of three independent clones were then spotted on semi-complete medium lacking tryptophane or adenine to assess plasmid curation and adenine auxotrophy (bottom). **(E) CD-FDp prion phenotype can be transmitted by cytoduction.** Donor CD-FD prion (1869) and [*URE3*] prion (287) strains were mated to a non-prion CD-FD recipient strain carrying a *kar-1* mutation (2112). After 7 h of mating, yeast cells were spread on selective N4W medium (glycerol as the carbon source and lacking adenine and uracil). Cells were incubated at 30 ${}^{\circ }$ C for one week. When donor cells contained prionized CD-FDp form (upper panel), small colonies appeared and were clearly identified under the binocular loupe (right part of panel e). In contrast, when donor cells contained prionized Ure2p form, only large colonies (indicated by the arrow) could be observed (lower panel). These large colonies were obtained with both donor strains and did not mate with a *MAT* α strain, unlike the small colonies obtained from the CD-FD prion donor. Each scale bar represents 1 cm.

### CD-FDp ${}^{I}$ can be transmitted by cytoduction

A *kar1* mutation delays nuclear fusion between the donor and recipient cells, allowing isolation of a cytoductant that inherits the nucleus of the recipient and the cytoplasm from both parents. “Canonical” yeast prions are situated in the cytosol, so cytoduction allows recipient strains to acquire these prion aggregates 22. We constructed a *MAT***a***kar1 ure2* Δ *ade2 pDal5::ADE2 lys2* Δ *::pGPD-CD-FD* recipient strain that was respiratory-deficient [*rho* ${}^{\circ }$ ], because it lacked mitochondrial DNA (mtDNA). Cytoductants inherit mitochondria from the donor and can be recognized as recipients that have become [*RHO* ${}^{+}$ ]. Two donor strains were used: CD-FD prion (pink, 1869), and [*URE3*] SB 34 23 as a negative control. Both donor strains are Ura ${}^{-}$ . After a short time, the mating mixture was spread on medium without adenine and uracil and with glycerol as the carbon source to obtain recipients that became [*RHO* ${}^{+}$ ] and Ade ${}^{+}$ (caused by acquiring the donor’s prion), as well as diploids. The plates were incubated for one week. We got hundreds of small colonies when the CD-FD prion strain was used as the donor. In contrast, none of these small colonies could be observed when the [*URE3*] strain was the donor (Figure 2**E**). Fifty of these small colonies were streaked on complete medium and tested for their capacity to grow without adenine and to mate. More than 90% were indeed *MAT***a** and Ade ${}^{+}$ indicating that they were recipients that acquired the Ade ${}^{+}$ phenotype during cytoduction. The non-mating small colonies were diploids. A few large non-maters also appeared that were clearly not cytoductants. An aliquot of the mating mixture was also spread on the same medium supplemented with adenine. Colonies appeared on these plates three times more frequently than in the absence of adenine. It thus appears that the CD-FDp prion was cytoduced along with mitochondria about one-third of the time.

### P53 core-domain prionization dependance on chaperones

Canonical yeast prions are dependent on Hsp104 24 for propagation during mitosis. This chaperone is necessary to break prion amyloid fiber, leading to the multiplication of propagons (prion’s transmissible entities). More recently, the existence of non-classical yeast prions which rely not on Hsp104 but on other chaperones like Hsp70 or Hsp90 was described 15. Thus, we tested the requirement of these different chaperones, for which there are known inhibitors, for the maintenance of CD-FDp prions. Hsp70 has a dominant-negative allele (*SSA1-21*) 25 and can also be inhibited by the anticancer drug pifithrin (PFT)- μ 26. Hsp90 has been reported to be inhibited by geldanamycin and radicicol. Importantly, most of the non-canonical prions (i.e., prion-like proteins without a Q/N-rich domain) isolated in yeast can be eliminated by these drugs and/or by the Hsp70 dominant-negative allele *SSA1-21* 15. Hsp104 is inhibited by guanidium chloride and cures canonical prions.

In two separate experiments, CD-FD white (1870) and pink (1869) prion strains were transformed with either an empty vector (pGPD) or a vector expressing the Hsp70 dominant-negative allele (pGPD-Hsp70 ${}^{\mathrm{DN}}$ ). As a control, we verified that the heat-shock treatment did not destabilize the prion phenotype (data not shown). For each strain, 12 independent transformants carrying either pGPD or pGPD-Hsp70 ${}^{\mathrm{DN}}$ were patched onto adenine-containing, plasmid-selective medium and incubated for 3 days. These were each repatched three times to allow for Hsp70 ${}^{\mathrm{DN}}$ expression and action on the prion before assessing adenine auxotrophy (Figure 3**A**). For the white prion strain, ten pGPD-Hsp70 ${}^{\mathrm{DN}}$ transformants remained Ade ${}^{+}$ , one became Ade ${}^{-}$ and one presented partial loss of the Ade ${}^{+}$ phenotype. In the control strain (pGPD transformants), eight remained Ade ${}^{+}$ , two became Ade ${}^{-}$ and two presented partial loss of the Ade ${}^{+}$ phenotype. For the pink prion strain, nine pGPD-Hsp70 ${}^{\mathrm{DN}}$ transformants remained Ade ${}^{+}$ , two became Ade ${}^{-}$ and one presented partial loss of the Ade ${}^{+}$ phenotype. In the control strain (pGPD transformants), five remained Ade ${}^{+}$ , five became Ade ${}^{-}$ and two presented partial losses of the Ade ${}^{+}$ phenotype. These results indicate that although the prion itself is not perfectly stable, Hsp70 ${}^{\mathrm{DN}}$ doesn’t enhance prion loss.

To confirm this result and challenge the role of other Hsp, we tested the impact of the chemical inactivation of Hsp70, Hsp90 and Hsp104 on the stability of CD-FDp inactivation. In two independent experiments, CD-FD non-prion strain (1844), CD-FD prion strains (1870 white and 1869 pink) and [*URE3*] prion strain (*MAT***a***trp1-1 ade2-1 leu2-3,112 his3-11,15 ura2* Δ *::HIS3 pDal5::ADE2*) were streaked on complete medium. A pool of clones was then streaked on complete medium supplemented or not with either guanidium chloride (GndHCl 5 mM), PFT- μ (50 μ M) or radicicol (50 μ M). After 3 days, a single isolated clone from these plates was subsequently streaked a second time under the same conditions (Figure 3**B**). As expected, in presence of GndHCl, the [*URE3*] strain became red, indicating an efficient curing. In contrast, none of the CD-FD prion strains were affected, indicating their capacities to propagate without active Hsp104. Some synthetic yeast prions depend on Hsp104 but failed to be efficiently cured by GndHCl 27. Disruption of *HSP104* was performed in strain 1870. As expected, *HSP104* disruption led to thermosensitive phenotype. Even in absence of Hsp104, this strain remained Ade ${}^{+}$ (Supplementary Figure S3). At the tested concentration, radicicol exhibited no prion-curing effect in either [*URE3*] or CD-FD prion strains. We observed the same results with PFT- μ . As we lack a positive control (but for GndHCl), we cannot exclude a possible effect at a higher concentration, or upon a longer drug exposition-time. On the other hand, increasing the drug concentration may also lead to cellular toxicity.

**Figure 3:**
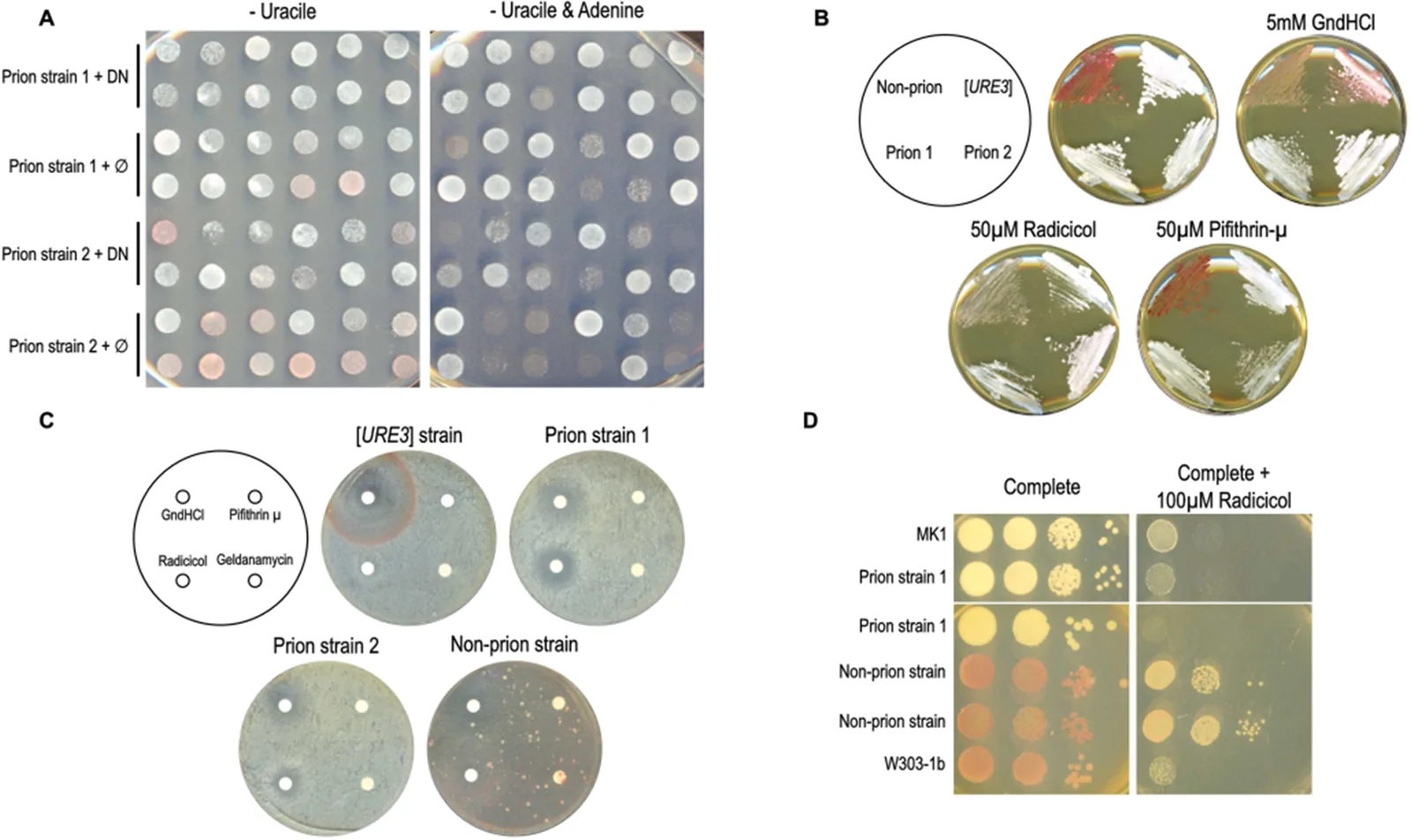
Curability of CD-FDp prion by chaperones inhibition. **(A) Hsp70 dominant negative allele does not cure CD-FDp prion.** CD-FD prion strain 1 (1870) and 2 (1869) were transformed either by an empty vector (pGPD, ∅ ) or a vector allowing the expression of a dominant-negative allele of Hsp70 (pGPD- Hsp70 ${}^{\mathrm{DN}}$ , DN), both carrying an *URA3* marker. Among the Ura ${}^{+}$ colonies, 12 independent transformants were streaked three times for 72 hours on plasmid-selective medium before being spotted on plasmid-selective medium (-uracile) supplemented or not with adenine. **(B) Chemical inhibition of Hsp70 or Hsp90 does not cure CD-FDp prion.** CD-FD non-prion strain, CD-FD prion strain 1 and 2 and an [*URE3*] strain were streaked two times for 72 h onto YPD plates supplemented with either 5 mM guanidine hydrochloride, 50 μ M Radicicol, or 50 μ M Pifithrin- μ , alongside an untreated control. **(C) CD-FDp prion confers radicicol sensibility.** 10 ${}^{6}$ cells of the CD-FD non-prion, CD-FD prion strain 1 and 2 and an [*URE3*] strain were spread on complete medium. On each plate are four filters soaked by 10 μ L of GndHCl (5 m), pifithrin- μ (10 mM), radicicol (5 mM), and geldanamycin (5 mM). **(D) CD-FDp confers resistance to radicicol**. The MK1, prion 1 (1870), non-prion (1844) and W303-1b (*MAT* α *leu2-3,112 trp1-1 can1-100 ura3-1 ade2-1 his3-11,15*) strains were streaked on complete medium. Isolated clones were serially diluted and spotted on complete medium and complete medium complemented with 100 μ M radicicol. Plates were observed after 3 days of incubation.

We subsequently used the antibiogram method, thus mimicking a drug concentration gradient. Around 10 ${}^{6}$ cells of each strain were spread on complete medium and 10 μ L of either 5 M GndHCl, 10 mM PFT- μ , 5 mM radicicol or 5 mM geldanamycin were spotted on filter disks placed on the petri dishes. After 3 days at 30 ${}^{\circ }$ C (Figure 3**C**), the expected halo of red colonies corresponding to cured cells was detected surrounding the spot of GndHCl when the [*URE3*] strain was spread on complete medium (Figure 3**C**). None of the other tested drugs induced prion curing in this strain. When white or pink prion strains were plated on complete medium, we didn’t observe any red halo. These results are consistent with our previous finding and confirm the lack of effect of these drugs on CD-FDp prion propagation at any concentration.

Interestingly, for both CD-FD prion strains, the cellular density was markedly reduced in the vicinity of the radicicol-spotted filter, indicating a toxic effect of this drug. The inhibition diameter was larger for the white prion strain 1 compared to pink prion strain 2, indicating a higher sensitivity to the drug. In the white prion strain, the concentration of active CD-FDp is expected to be lower than in the pink strain (the biosynthetic pathway of adenine is more derepressed) which might indicate that somehow, CD-FDp has a protective effect. Alternatively, the prionization of CD-FDp may exacerbate the sensitivity to radicicol. To test this hypothesis, we spotted serial dilutions of different strains on complete medium complemented with 100 μ M radicicol (Figure 3**D**). The MK1 strain shares the same background as the CD-FD prion and non-prion strains but does not carry the pGPD-CD-FD cassette. The W303-1b strain (*MAT* α *leu2-3,112 trp1-1 can1-100 ura3-1 ade2-1 his3-11,15*) has a different genetic background but expresses functional Ure2p and shares the Ade ${}^{-}$ phenotype with the CD-FD non-prion strain. Both the W303-1b and MK1 strains are susceptible to radicicol, as is the CD-FD prion strain. However, the CD-FD non-prion strain remained resistant to the drug. Thus, it is not the presence of CD-FDp prion which confers radicicol sensitivity but rather the expression of functional CD-FDp which leads to radicicol resistance. As W303-1b expresses Ure2p, we believe that the resistance is not related to Ure2p activity, but rather to the expression of the p53 core-domain. In that case, the p53 core-domain alone or a fusion between this domain and another functional domain should behave similarly, but this was not tested.

The ability of CD-FDp ${}^{I}$ to be transmitted efficiently during mitosis independently of so many chaperones is quite unusual. The two archetypal yeast prions, [*URE3*] and [*PSI* ${}^{+}$ ], are cured in the absence of active Hsp104 28, 29. A new category of prion phenotype, based on large non-Q/N-rich intrinsically disordered domains, was found to be independent on Hsp104, but is often sensitive to the expression of other chaperones 15. In *S. cerevisiae*, p53 has been shown to behave as a prion whose transmission from mother to daughters cells does not require active Hsp104 or Hsp90 14. Since CD-FDp is derived from the core-domain of p53 and both p53 and CD-FDp prions share these same properties, we hypothesized that there may be a cross-talk in the prionization of these proteins.

### Prionization of CD-FDp echoes full-length p53 (FLp53) prionization

To determine if prionized CD-FDp can induce the formation of P53-EYFP foci, as is the case for prionized p53 14, CD-FD prion and non-prion strains were transformed with pGPD-p53-EYFP, allowing the constitutive expression of p53 full-length (FLp53) tagged with EYFP. Six independent transformants of pink prion *MAT***a** and *MAT* α (1868 and 1869 respectively), white prion *MAT***a** and *MAT* α (1870 and 1871 respectively), non-prion *MAT***a**, *MAT* α and 2n (1851, 1844 and 1855 respectively), were grown for 2 days and observed for fluorescence. All strains had diffuse signal in the cytoplasm and some cells showed diffuse nuclear staining as well. We looked for punctate cytoplasmic foci corresponding to aggregated p53 (Figure 4**A**). The prion strains exhibited from 6.4 to 11.9% of cells with foci, while non-prion strains showed 2.2 to 5% of cells with foci. On average, 8.6% of prion strain cells presented cytoplasmic foci compared to 3.8% of non-prion strain cells (Figure 4**B**). Notably, a substantial fraction of non-prion cells also displayed cytoplasmic p53-EYFP foci, indicating that p53-EYFP aggregation is not strictly dependent on the prion state. All four prion strain averages exceeded all three non-prion strain averages. Since only four and three independent strains strains were examined, the lowest two-sided p-value obtainable from an exact Mann-Whitney U test even under complete separation of the two groups is 0.057. This is the p-value revealed by our data and while consistent with a difference between prion and non-prion strains, it does not reach statistical significance at the 0.05 conventional threshold. However, a one-sided test restricted specifically to the hypothesis that prion strains show a higher percentage of foci-positive cells yields p=0.029 . This result is consistent with chimeric protein inactivation due to aggregation, although less efficiently than prionized FLp53 (6.4–11.9% vs. 15–30%) 14.

Yeast prions form cytoplasmic amyloid aggregates that can be stained with Thioflavin T (ThT). This is also the case for prions formed by p53 14. We tested the capacity of prionized CD-FDp to induce such aggregates (Figure 4**C**). As a control, five independent prion subclones of L3672 and two independent subclones of non-prion L3671 strains expressing FLp53 were fixed and the presence of foci detected by ThT fluorescence was scored in three independent clones for each strain. The percentage of cells with aggregates varies from 1.1 to 5.7% for the non-prion strains and from 8.2 to 18.5% for the prion strains. Averaged, this amounts to 3.1% in the non-prion vs 12.2% in the prion strain (Figure 4**D**) and this difference is statistically significant (Student’s t-test, p-value = 0.01151).

**Figure 4:**
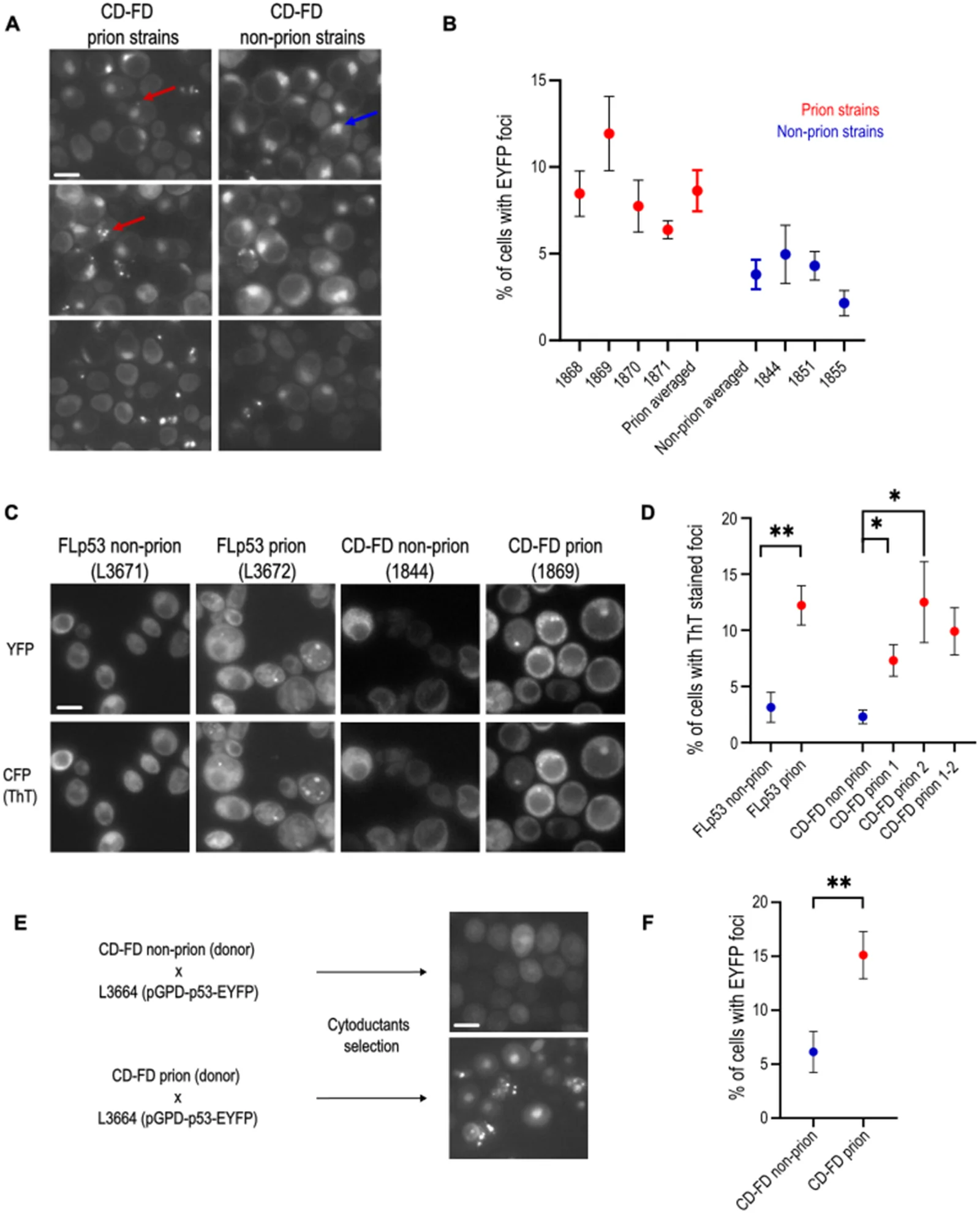
Cross-talk between full length p53 (FLp53) and CD-FDp. **(A) CD-FDp prion can induce FLp53 cytoplasmic foci (qualitative analysis).** Four prion and three non-prion strains were transformed with pGPD-p53-EYFP (*HIS3, CEN*), patched on dextrose-based plasmid-selective medium and grown for 2 days before imaging with a YFP filter cube. Three representative images for the prion and non-prion strains are presented. Scale bar: 5 μ m. Examples of cytoplasmic foci and nucleus diffuse signal are respectively marked with red and blue arrows. **(B) CD-FDp prion can induce FLp53 cytoplasmic foci (quantitative analysis)**. For all prion (1868, 1869, 1870, 1871) and non-prion (1844, 1851, 1855) strains, the number of cells with YFP fluorescence examined as well as the number of cells presenting cytoplasmic foci were counted. Each YFP fluorescence observation was performed on 80–400 cells. Percentage of cells presenting cytoplasmic p53-EYFP foci are represented within standard error of the mean (SEM). SEM was determined based on six biological replicates for each strain and represented by error bars. The four prion and three non-prion strain averages were compared, to test whether they represent statistically distinct groups, using an exact two-sided Mann-Whitney U test ( p=0.057 ) (see text). **(C) Cytoplasmic foci are amyloid-like (qualitative analysis).** FLp53 prion L3672 subclones and non-prion L3671 subclones as well as CD-FD prion (1869, 1871) and non-prion (1844) strains were transformed with pGPD-p53-EYFP (*HIS3, CEN*). Three independent transformants of each strain were grown on dextrose plasmid selective medium to express p53-EYFP. Cells were harvested, fixed, stained with Thioflavin T (ThT) and examined by fluorescence microscopy. Representative images showing p53-EYFP fluorescence (YFP filter cube) and ThT staining (CFP filter cube) are shown. Scale bar: 5 μ m. **(D) Increased amyloid signal in CD-FD prion strains (quantitative analysis).** The total number of cells examined and the number of cells displaying ThT-positive foci were counted. The percentage of cells with ThT-stained foci was calculated and averaged across three biological replicates per strain. CD-FD non-prion strain was compared to each of the CD-FD prion strains as well as to the two prion strains combined. Statistical significance was determined using Student’s t-test, and error bars represent the standard error of the mean. p-value < 0.05: ${}^{*}$ , p-value < 0.01: ${}^{**}$ **. (E) The prion phenotype - inducing element is cytoplasmic (qualitative analysis).** CD-FD (*MAT* α , [*RHO* ${}^{+}$ ]) non-prion and prion strains were used as donor to cytoduction recipient L3664 (*MAT***a**, [*rho* ${}^{\circ }$ ], *kar1*) transformed with pGPD-p53-EYFP (*HIS3, CEN*) (Left). Six cytoductants obtained from the non-prion strain and 10 obtained from the prion strain were assayed for EYFP fluorescence with a YFP filter cube. One representative image for each cytoduction is presented (Right). Scale bar: 5 μ m. **(F) The prion phenotype - inducing element is cytoplasmic (quantitative analysis).** The number of cells examined as well as the number of cells with cytoplasmic foci were counted, and the ratios were averaged and compared using Student’s t-test. Standard error of the mean was determined based on the biological replicates (150–400 cells each of 6 independent non-prion and 10 independent prion cytoductants) and represented by error bars. p-value < 0.01: ${}^{**}$ .

To examine whether p53-EYFP aggregates formed in CD-FD prion strains exhibit amyloid-like properties, we analyzed ThT staining in cells expressing p53-EYFP in both prion (pink 1869 and white 1871) and non-prion (1844) backgrounds (Figure 4**C**). Transformants expressing pGPD-p53-EYFP were grown for 2 days on plasmid selective plates, fixed, and stained with ThT. The presence of ThT-positive foci was scored by fluorescence microscopy in three independent clones per strain. In the non-prion strain (1844), the percentage of cells showing ThT staining ranged from 1.5 to 3.4%, with an average of 2.3%. In contrast, prion strain pink 1 (1869) exhibited 5.0 to 9.9% ThT-positive cells, averaging 7.3%, while prion strain white 2 (1871) showed higher levels ranging from 8.7 to 19.8%, with an average of 12.5%. When the two prion strains were combined, the overall percentage of ThT-positive cells in prion backgrounds averaged 9.9%, compared to 2.3% in the non-prion strain (Figure 4**D**). Statistical analysis revealed that the increase in ThT-positive cells in prion strains was significant when compared to the non-prion control (Student’s t-test; non-prion vs prion 1, p-value = 0.0301; non-prion vs prion 2, p-value = 0.0486; non-prion vs combined prion strains, p-value = 0.0428). Consistent with the microscopy images, ThT fluorescence mostly co-localized with p53-EYFP foci in both strains.

Together, these results demonstrate a clear correlation between the prion state and increased ThT staining in cells expressing p53-EYFP, indicating that p53 aggregates formed in CD-FD prion strains are enriched in amyloid-like structures. This supports the conclusion that prionization of CD-FDp enhances amyloid aggregate formation of p53 in yeast.

Since prionization of p53 and CD-FDp both led to the formation of p53-EYFP foci and to an increase of ThT-binding aggregates, we tested the capacity of the CD-FDp ${}^{I}$ to prionize p53 *via* cytoplasmic information only. Cytoduction was used to transfer cytoplasm from *MAT* α [*RHO* ${}^{+}$ ] prion (1869 and 1870) and non-prion (1844) CD-FD strains to a *MAT***a***kar1* [*rho* ${}^{\circ }$ ] (L3664) recipient strain transformed with the pGPD-p53-EYFP plasmid 14. We isolated cytoductants by selecting for recipient cells that could perform respiration and had appropriate markers excluding donors and diploids (see Materials and Methods). Six cytoductants obtained with non-prion and ten obtained with the prion strain donors were examined for cytoplasmic foci (Figure 4**E**). 6.1% of cells from cytoductants with a non-prion donor and 15.1% of cells from cytoductants with a prion donor had foci (Figure 4**E**). This difference in mean percentages is statistically significant (Student’s t-test, p-value = 009781). This result suggests that the cytosol of prionized CD-FD strains possess conformational information that can be transmitted to FLp53. Once transmitted, this conformational information may propagate during mitosis independently of the expression of CD-FDp. These observations fit a model in which propagons from a CD-FD prion strain can switch functional p53 into a prion conformation as previously reported for propagons made from p53.

Thus, the p53 core-domain prions can drive p53 full length prionization and could represent a valuable model to quantify the prionization propensity of p53.

### CD-FDp inactivation frequency varies with cancer-associated mutations

Over 80% of cancer-associated p53 mutations occur in the core-domain of p53, and some of these mutations are associated with aggregation-prone capacity. In order to quantify this propensity with the yeast system, we measured the CD-FDp inactivation frequency using a Luria-Delbruck fluctuation assay 30–32. First, we performed the analysis with ectopic expression of the fusion protein. MK1 (*ure2* Δ *::cyh2 trp1-1 ade2* Δ *::Kan* ${}^{R}$ *pDal5::ADE2*) was transformed with pGPDH-CD-FD and transformants were selected on synthetic plasmid-selective medium. In two independent transformations, six clones were tested for CD-FDp inactivation frequencies (Figure 5**A**). The number of Ade ${}^{+}$ colonies was determined after 3 days of incubation at 30 ${}^{\circ }$ C (supplementary data table S1). This number results from both the frequency of inactivation events and the number of generations that have passed since their occurrence (Figure 5**B**). To extract inactivation frequency per generation from these data, we used an algorithm based on maximum likelihood 33. We observed inter-clone variability with a CD-FDp inactivation frequency varying from 5.91 × 10 ${}^{-8}$ to 7.33 × 10 ${}^{-7}$ per generation (Figure 5**C**, Supplementary table S2). Altogether, the data indicate an inactivation frequency of 2.14 × 10 ${}^{-7}$ per generation (95% confidence interval: 1.77 × 10 ${}^{-7}$ – 2.55 × 10 ${}^{-7}$ ).

We subsequently asked whether cancer-associated p53 mutations would influence the CD-FDp inactivation frequency. In human tumors, p53 wild-type has been found to be aggregated 34, 35. Some variants have been identified as more aggregation-prone than the wild-type protein 1. We cloned five alleles of the p53 core-domain in frame with the Ure2p functional domain into the pGPDH-CD-FD vector: R248W-C142T, R273H, R249S, R282W (cDNA kindly provided by R. Iggo) and R175H. These variants are the result of hotspot mutations, commonly found in cancer patients (with the exception of the likely passenger mutation C142T, which is not described in the p53 variants database). For each variant, five to eight independent transformants were analyzed. We counted Ade ${}^{+}$ colonies (supplementary data table S3). Inactivation frequencies within their lower and upper limit (95% confidence interval) are presented in Figure 5**D**. These frequencies were compared to the wild-type allele’s using the Mandelbrot-Koch comparison. The inactivation frequencies of R273H and R282W mutants were not statistically different from the wild-type (Mandelbrot-Koch, p-value > 0.05), whereas the R248W-C142T mutant exhibited a 1.33-fold increased likelihood of inactivation (Mandelbrot-Koch, p-value = 1.3856 × 10 ${}^{-2}$ ) and R249S a 1.75-fold increased likelihood of inactivation (Mandelbrot-Koch, p-value = 4.5157 × 10 ${}^{-6}$ ).

**Figure 5:**
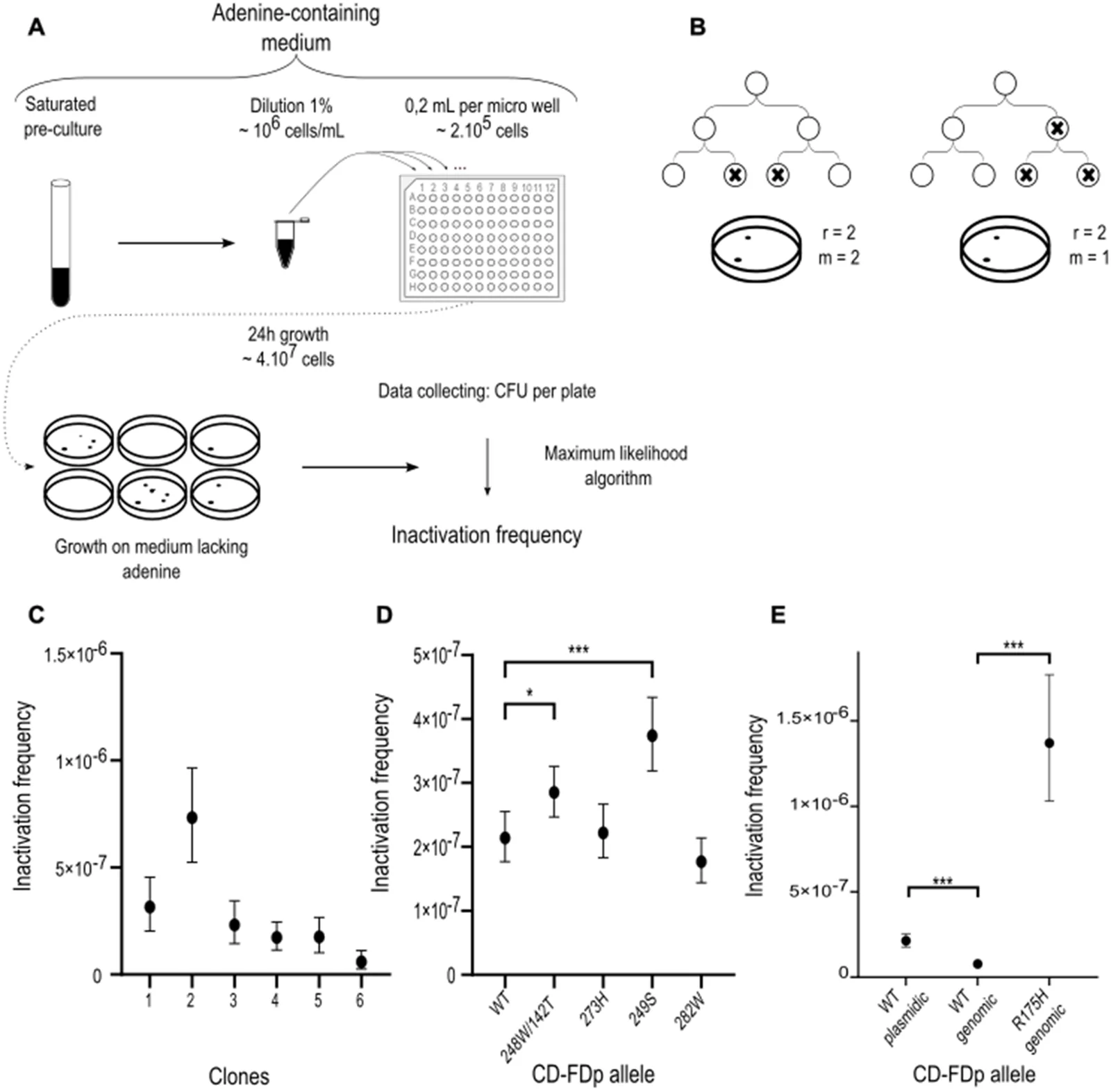
Inactivation frequency is driven by CD sequence and expression level. **(A) Luria Delbruck’s fluctuation assay.** A CD-FDp expressing clone was cultivated in adenine-containing medium until saturation. The culture was diluted 1:100 in 16 wells of a microplate, each containing 200 μ L of the same medium. Each well contains approximatively 2 × 10 ${}^{5}$ cells, a number below the estimated inactivation frequency of CD-FDp, thereby reducing the likelihood of incubating cells in which CD-FDp is already inactivated. Those micro-cultures were grown until saturation, allowing CD-FDp to switch from active to inactive. They were finally spread onto a medium lacking adenine. After 3 days, Ade ${}^{+}$ cells were counted and the number of Ade ${}^{+}$ colonies for each culture treated through a maximum likelihood algorithm to deduce inactivation frequency per generation. **(B) Inactivation event vs CD-FDp – inactive clones.** If two Ade ${}^{+}$ clones are counted ( r=2 ), they could be resulting either from two independent inactivation events ( m=2 , left) or from a unique inactivation event ( m=1 , right). The maximum likelihood algorithm allows estimation of inactivation event frequency per generation from the number of cells in which CD-FDp has been inactivated in 16 independent micro-cultures. **(C) Inactivation frequency of CD-FDp expressed from a plasmid is 2.14 × 10** ${}^{\mathrm{-7}}$ **per generation.** MK1 (*ure2* Δ ) was transformed with pGPDH-CD-FD. In two independent transformations, we selected six clones and measured their inactivation frequency. Estimated inactivation frequency are represented within their 95% confidence interval. **(D) Inactivation frequency of CD-FDp is influenced by cancer-associated mutation.** MK1 was transformed with mutated pGPDH-CD*-FD. Among the His ${}^{+}$ clones obtained, 5 to 8 independent transformants of each CD-FDp allele were tested and their inactivation frequency were measured using fluctuation assay. Estimated inactivation frequency are represented within their 95% confidence interval and statistical analysis was done using Mandelbrot-Koch comparison. WT: wild-type, p-value < 0.05: ${}^{*}$ , p-value < 0.01: ${}^{**}$ , p-value < 0.001: ${}^{***}$ **. (E) CD** ${}^{\mathrm{R175H}}$ **-FDp is 17.7 times more likely to be inactivated than wild-type CD-FDp.** We used three CD-FD subclones and two CD ${}^{\mathrm{R175H}}$ -FD subclones in which CD-FD is integrated at the *LYS2* locus to measure their inactivation frequency using fluctuation assay. Inactivation frequencies of CD-FDp expressed from 2 μ plasmid is also shown. Estimated inactivation frequency are represented within their 95% confidence interval and statistical analysis was done using Mandelbrot-Koch comparison. WT: wild-type, p-value < 0.05: ${}^{*}$ , p-value < 0.01: ${}^{**}$ , p-value < 0.001: ${}^{***}$ .

The R175H variant led to an uncountable number of Ade ${}^{+}$ colonies for all micro-cultures. This result indicates a very high propensity for the chimeric protein to become inactivated and did not allow us to measure its inactivation frequency. The plasmid used for these experiments has a 2 μ replication origin, leading to a high number of plasmid copies per cell, and therefore to a high concentration of cellular CD-FDp. It is well established that for [*URE3*] and [*PSI* ${}^{+}$ ], the level of expression of the prion proteins increases the frequency of prion formation 13, 19. To reduce protein expression, we integrated the transcriptional cassette allowing expression of CD ${}^{\mathrm{R175H}}$ -FDp at the *LYS2* locus and compared its inactivation frequency with wild-type integrated CD-FD (non-prion strain 1844). For this strain (integrated wild-type CD-FD), the analysis made with three independent clones indicates an inactivation frequency of 7.8 × 10 ${}^{-8}$ per generation (Figure 5**E**, supplementary data table S4), which represents a 0.36-fold change compared to plasmidic expression of wild-type CD-FDp (7.8 × 10 ${}^{-8}$ vs 2.14 × 10 ${}^{-7}$ per generation for plasmidic expression, Mandelbrot-Koch, p-value = 2.9852 × 10 ${}^{-9}$ ). The CD ${}^{\mathrm{R175H}}$ -FDp inactivation frequency determined with two independent clones is 1.4 × 10 ${}^{-6}$ per generation. This represents a 17.7-fold change compared to the wild-type CD-FDp (Mandelbrot-Koch, p-value < 10 ${}^{-9}$ ). This huge increase in inactivation frequency echoes the difference in aggregation propensity between the wild-type and the R175H proteins 36, 37.

## DISCUSSION

In this study, we demonstrated that the p53 core-domain (p53CD) can function as a prion-forming domain. Using a genetic system based on a chimeric Ure2 protein (where the native prion-forming domain is replaced by p53CD (CD-FDp)), we found that inactive CD-FDp exhibited a higher propensity to form larger species recoverable in the pellet after ultra-centrifugation. Notably, CD-FDp inactivation is dominant, transmissible by cytoduction and displays non-Mendelian segregation, all hallmarks of prion behavior.

### P53CD prion domain begets prions strains

With the [*URE3*] system using the same *pDal5::ADE2* reporter as the strains used in this study, Ade ${}^{+}$ prion-bearing colonies can appear white or pink 38. Our results show that CD-FD Ade ${}^{+}$ colonies exhibit the same color variability.

We identified two phenotypic classes: white (stable propagation) and pink (converts to white upon serial passage). White color is associated with the stronger activation of the AMP pathway, *i.e.*, the lower amount of active CD-FDp obtained that correspond to the more efficient sequestration of CD-FDp. We consider these two phenotypic classes as candidate prion variants, while acknowledging that the small number of tetrads analyzed precludes definitive conclusions about their nature and frequency.

The asymmetry in stability between white and pink is consistent with competitive dynamics among prion variants, where more stable conformations are selectively amplified over time. The appearance of both phenotypic classes from a single selected diploid may reflect pre-existing structural variants in the diploid cell, with pink representing a distinct but less competitive prion variant. Following segregation of the variants during mitotic replication of the diploid, cells with either one or the other variant could arise. Under this model, sporulation of variant-mixed diploid cells would be expected to produce asci in which all prion-bearing spores share one of the variants in the parent diploid, as we observed. Alternatively, pink could represent a transitional state that resolves to white, similar to early [*PSI* ${}^{+}$ ] maturation, where multiple variants were shown to be induced in a single cell that matured into one variant within a few generations 39. This model is also broadly consistent with within-ascus uniformity, if conformational resolution occurred at the diploid cell level prior to meiosis.

That the CD-FDp gives rise to at least two phenotypically distinguishable prion states supports the view that the p53 prion domain retains sufficient conformational flexibility to adopt more than one self-propagating architecture, though further work will be needed to determine whether these states represent structurally distinct variants in the classical sense. These findings are consistent with a system where the propagative species is amyloid-based.

### Chaperone independence: a distinct behavior from classical and atypical yeast prions

For the archetypes of yeast prions such as [*URE3*], propagons resulting from amyloid stacking tend to form larger aggregates that can ultimately sequester all the propagons, leading to their retention in mother cell 40, 41. The balance between soluble propagons and larger aggregates is tuned by Hsp104, which plays a crucial role in prion propagation. However, Hsp104 is not required for the maintenance of CD-FDp inactivation, consistent with observations for full-length p53 (FLp53). This independence is shared with another category of prion-like proteins, which rely on intrinsically disordered sequences lacking the Q/N-rich signature 15. Most of these determinants are dependent on Hsp70 and Hsp90 activities. However, CD-FDp does not require any of these chaperones for propagation, distinguishing it from both typical and atypical yeast prions.

Prionized CD-FDp lead to FLp53-EYFP foci-formation in approximately 10% of cells, yet prionized CD-FDp is propagated in the vast majority of cells. This discrepancy suggests that visible foci are not *bona fide* propagons entities, but may also reflect our incapacity to identify all the foci in the yeast cells. This aligns with previous findings by the authors 14, who observed foci in ∼ 30% of cells when FLp53 was prionized. Additionally, the ability of p53-EYFP aggregates to bind the amyloid dye ThT suggests an enrichment of amyloid-like structures in yeast cells propagating prionized CD-FDp.

Collectively, these results support a model in which CD-FDp prionization is linked to an autocatalytic aggregation process, which can subsequently be transmitted to full-length p53. This transfer of structural information can, in some cases, induce the formation of foci with amyloid-like structures.

### Can the yeast system predict pathogenic behavior?

P53 core-domain behaves like an atypical yeast prion domain, yet is structurally completely unrelated to the already known prion-like domains, which are typically intrinsically disordered. This is also true for the prion protein PrP, where prionization involves a structural shift from helical to beta-sheet-rich conformations. The p53 core-domain is highly structured 42 and correctly folded in yeast when not in the prion form since FLp53 is functional in this organism.

To assess whether the yeast system could predict the pathogenic behavior of p53 mutants, we analyzed a panel of clinically relevant mutations, including both “contact” mutants (R248W 42 and R273H 42, 43), which disrupt DNA binding without affecting stability, and “structural” mutants (R175H, R282W and R249S) which destabilize the core-domain 44. R175H, the most frequent hotspot mutation in cancer, is the canonical reference for studying conformational/structural inactivation of p53.

The genetic system described in this article allows for the accurate quantification of CD-FDp inactivation. While mechanisms other than prionization can also lead to inactivation, these would produce a constant background regardless of whether the p53 core-domain is mutated. By comparing the inactivation frequency of wild-type CD-FDp with that of mutated CD-FDp, we can assess the intrinsic relative capacity of the mutated domain to undergo prionization.

For the R175H mutant, we observed an ∼ 18-fold increase in the frequency of CD-FDp inactivation, a dramatic effect that correlates well with its destabilization of the core-domain. This mutant also exhibits a higher propensity to form cytoplasmic punctate when over expressed in mammalian cells 1. In contrast, the R273H mutation (a “contact” mutant) and R282W did not increase the inactivation rate. This is expected for R273H, which displays similar stability to wild-type 44. However, R282W destabilizes the p53 core-domain as effectively as R175H in reversible urea denaturation assays 45, suggesting it should be equally efficient at promoting unfolding. CD-FDp prionization involves not only the destabilization of the folded structure, but also the capacity to form a self-perpetuating alternative structure. Possibly, while R282W is inherently unstable, it does not go on to form a heritable prion-like particle. The R249S and R248W mutants, which are less stable than wild-type 46, 47 but more stable than R175H, exhibited only a modest effect (less than 2-fold increase in inactivation likelihood). This result could also have been influenced by slight variation in protein expression. For all mutants except R282W, *in vitro* stability correlates with the frequency of inactivation, opening the possibility of experimentally predicting prion-like behavior.

## CONCLUSION

This study establishes the p53 core-domain as a novel, structurally distinct prion-forming domain and demonstrates that the yeast system can quantify and predict the prion-like behavior of p53 mutants. These findings deepen our understanding of prion biology and provide a powerful experimental framework for studying the pathogenic mechanisms of p53 inactivation.

Furthermore, this system enables the screening of drugs or genes that can inhibit the prionization mechanism, potentially offering alternative therapeutic strategies to counteract p53 inactivation in cancer. Future work should focus on elucidating the structural basis of CD-FDp prion strains and exploring the therapeutic potential of targeting p53 prionization in human diseases.

## MATERIALS AND METHODS

### Growth media

Yeast strains (listed in Table 3 below) were routinely grown in YPD (yeast extract 10 g/L, peptone 20 g/L, glucose 20 g/L in distilled water). Auxotrophic selection was achieved on synthetic drop-out medium (Complete Supplement Mixture (CSM)-ADE-HIS-TRP-URA (Formedium) 740mg/L or CSM-HIS-TRP-URA-LEU (Formedium) 600mg/L), YNB (DIFCO) 6.7g/L, glucose 20 g/L, agar 20 g/L) supplemented when required with histidine, uracil, tryptophan, adenine (20 mg/L) and leucine (60 mg/L). Poor nitrogen medium (SDP) used proline as nitrogen source (YNB without amino acids and without ammonium 1.7 g/L, glucose 20 g/L, proline 1 g/L, agar 20 g/L).

Meiosis was induced by plating diploid cells on SP1 medium (potassium acetate 10 g/L, yeast extract 1 g/L, glucose 0,5 g/L, agar 20 g/L) complemented with necessary bases and amino acids at 1/10 ${}^{\mathrm{th}}$ concentration. The presence of tetrads was checked under the microscope after 2 days. After removal of the ascus membrane, spores were either separated with a Singer apparatus for tetrad analysis or physically separated from unsporulated cells by hydrophobic separation 48 and plated.

For cytoduction experiments, cytoductants were selected on N4W (YNB (DIFCO) 6.7g/L, casamino acids 6 g/L, glycerol 30 mL/L, agar 20 g/L, tryptophane 20 mg/L) or N4WA (N4W supplemented with adenine 20 mg/L).

### Transformation

Transformed strains were obtained by transforming 100 ng of plasmids with an optimized LiAc-based method 49.

### Mating and diploid selection

Unless specified otherwise, *MAT***a** strains were transformed with pAG423-Gal-ccdB (Addgene) (*HIS3*) and the *MAT* α strain with pAG424-GAL1-ccdB-eGFP (Addgene) (*TRP1*). *MAT***a** are therefore His ${}^{+}$ , Trp ${}^{-}$ and *MAT* α are His ${}^{-}$ , Trp ${}^{+}$ . Both transformants were mixed on complete solid medium for 6 h and replicated on semi-complete medium lacking histidine and tryptophane for diploid selection.

### Chimeric CD-FD plasmids

Plasmids used in this study are listed in Table 2 below. pGPDW-URE2 was obtained by cloning *URE2* ORF into pAG424-GPD-ccdB (Addgene) by Gateway 50. The p53 core-domain sequence was amplified from pLS76 51 with oligonucleotides 1242 and 1243 (see Table 1). After co-transformation of pGPDW-URE2 digested with NotI and BamHI and the PCR product, the plasmid was repaired and circularized by homologous recombination leading to Trp ${}^{+}$ colonies. Plasmids from Trp ${}^{+}$ transformants were analyzed by restriction and verified by sequencing to get pGPDW-CD-FD (811). In this plasmid, the *URE2* prion domain sequence (Nter) spanning positions 1 to 62 is replaced by the p53 core-domain sequence (spanning amino acids 61 to 307). pGPDH-CD-FD with the *HIS3* selectable marker (810) was obtained by exchanging the SacI-MluI fragment containing the *GAL* promoter in pAG423-Gal-ccdB (809) with the SacI-MluI fragment of pGPDW-CD-FD (811).

Mutated core-domains were amplified by PCR from yeast expression plasmids 53. These plasmids allow expression of the full-length p53 with mutations documented in human tumors. The protocol used was the same as the one to get pGPDW-CD-FD but pGPDH-CD-FD was used as backbone. All the plasmids were sequenced to verify the presence of the expected mutations. With this approach, we could get pGPD-R248W-FD (834), pGPD-R273H-FD (835), pGPD-R249S-FD (836) and pGPD-R282W-FD (837). Sequence analysis of the R248W mutant revealed an additional mutation found in initial plasmid (C142T). pGPD-R175H-FD was obtained by site-directed mutagenesis of wild-type pGPDH-CD-FD (810) using an optimized protocol based to the standard “Quikchange ${}^{\mathrm{Tm}}$ ” approach 54 with oligonucleotides 1272 and 1273.

**Table 1:** Oligonucleotides used .

**Oligo-ID**	**Sequence**
1189	cgttacagctacctcagctcgatgtgcctcatgataggctggagctcttcgagtttatc
1190	gctgctgcggagcttccacgagcaccagcacctgcaaattaaagccttcgagcgtcc
1242	cgttatcattattttggctaccattgcggccgcttgctcgcttagtgctccctggg
1243	cttagtttcgacggattctagaactagtggatccatggaagctcccagaatgccagagg
1272	gggggcagtgcctcacaacctccgtcatgtgctgtgactgcttgtag
1273	gtgaggcactgcccccaccatgagcgctgcagcgatagcgatggtctgg
1195	cagcccatcaccactttgtacaagaaagctgggtgtcagtgatggtgatggtgatgagc
1196	ctggccgcatcttctcaaatatgcttcccagcctgcgatccgtcgagttcaagag
1197	taaaacctctcgagaatattttgttgca
1198	atgatccaacaaaatattctcgagaggt
1199	atgatctgaaagagtaaccattgttggt
2000	taaaaccaacaatggttactctttcaga

**Table 2:** Plasmids used .

**Lab name and number**	**Original name and description**	**Reference**
pCM188-URE2 (260)	pCM-188-URE2 (*CEN,URA3*)	52
pGPD (672)	pAG424-GPD-ccdB (2 μ , *TRP1*)	Addgene plasmid #14152
pGPDW-URE2 (685)	pAG424-GPD-URE2 (2 μ *, TRP1*)	This study
TEF1p-Cas9 (745)	p414-TEF1p-Cas9-Cyc1t (*CEN, TRP1*)	Addgene plasmid #43802
p426-SNR52p-gRNA (746)	p426-SNR52p-gRNA.CAN1.Y-SUP4t (*2* μ *, URA3*)	Addgene plasmid #43803
gRNA1-Lys2 (755)	P426gRNA1-Lys2 (*2* μ *, HIS3*)	This study
gRNA2-Lys2 (756)	P426gRNA2-Lys2 (*2* μ *, HIS3*)	This study
pADH1-p53 (808)	pADH1-p53 (*CEN, LEU2*)	50
pGal-ccdB (809)	pAG423-Gal-ccdB (*2* μ *, HIS3*)	Addgene plasmid #14149
pGPDW-CD-FD (811)	pAG424-GPD-p53CD-URE2FD (*2* μ *, TRP1*)	This study
pGPDH-CD-FD (810)	pAG423-GPD-p53CD-URE2FD (*2* μ *, HIS3*)	This study
pGal-ccdB-eGFP (818)	pAG424-GAL1-ccdB-eGFP (*2* μ *, TRP1*)	Addgene plasmid #14199
pGPD (825)	pAG426GPD-ccdB (*2* μ *, URA3*)	Addgene plasmid #14156
pGPD-Hsp70 ${}^{\mathrm{DN}}$ (876)	pAG426-GPD-SSA1 ${}^{\mathrm{DN}}$ K69M (*2* μ *, URA3*)	Caudron’s lab
pGPD-p53-EYFP	pGPD1-p53-EYFP (*CEN, HIS3*)	14
pRI-273H (829)	pADH1-p53 ${}^{\mathrm{R273H}}$ (*CEN, LEU2*)	51
pRI-282W (830)	pADH1-p53 ${}^{\mathrm{R282W}}$ (*CEN, LEU2*)	51
pRI-248W-142T (831)	pADH1-p53 ${}^{\mathrm{R248W/C142T}}$ (*CEN, LEU2*)	51
pRI-249S (832)	pADH1-p53 ${}^{\mathrm{R249S}}$ (*CEN, LEU2*)	51
pGPD-CD ${}^{\mathrm{R175H}}$ -FD (845)	pAG423-GPD-CD ${}^{\mathrm{R175H}}$ -FD (*2* μ *, HIS3*)	This study
pGPD-CD ${}^{\mathrm{248W}}$ -FD (834)	pAG423- GPD-CD ${}^{\mathrm{R248W/C142T}}$ -FD (*2* μ *, HIS3*)	This study
pGPD-CD ${}^{\mathrm{273H}}$ -FD (835)	pAG423- GPD-CD ${}^{\mathrm{R273H}}$ -FD (*2* μ *, HIS3*)	This study
pGPD-CD ${}^{\mathrm{249S}}$ -FD (836)	pAG423- GPD-CD ${}^{\mathrm{R249S}}$ -FD (*2* μ *, HIS3*)	This study
pGPD-CD ${}^{\mathrm{282W}}$ -FD (837)	pAG423- GPD-CD ${}^{\mathrm{R282W}}$ -FD (*2* μ *, HIS3*)	This study

**Table 3:** Strains used .

**Strain name and lab number**	**Description**	**Reference**
BY4742 *ade2* Δ (1836)	*MAT* α *his3* Δ *1 leu2* Δ *0 lys2* Δ *0 ura3* Δ *0 ade2* Δ *::kanMX*	EUROSCARF knockout library
BY188 (1589)	*MAT***a***ade2 his3 leu2-3,112 ura2* Δ *ure2* Δ *pDal5::kanMx pDal5::Can1 kar1*	55
NT62 (816)	*MAT***a***trp1-1 ade2-1 his3-11,15 leu2-3,112 ura3-1 pdal5::ADE2 ure2* Δ *::cyh2*	
MK1 (1840)	*MAT***a***his3* Δ *1 leu2-3,112 ura3-1 trp1-1 ade2* Δ *::kanMX pDal5::ADE2 ure2* Δ *::cyh2*	This study
MK2 (1842)	*MAT* α *his3* Δ *1 leu2-3,112 ura3-1 trp1-1 ade2* Δ *::kanMX pDal5::ADE2 ure2* Δ *::cyh2*	This study
CD-FD (1851)	*MAT***a***his3* Δ *1 leu2-3,112 ura3-1 trp1-1 ade2* Δ *::kanMX pDal5::ADE2 ure2* Δ *::cyh2 lys2* Δ *::pGPD-CD-FD*	This study
CD-FD (1844)	*MAT* α *his3* Δ *1 leu2* Δ *0 ura3-1 trp1-1 ade2* Δ *::kanMX pDal5::ADE2 ure2* Δ *::cyh2 lys2* Δ *::pGPD-CD-FD*	This study
CD-FD 2n (1855)	*MAT***a***/* α *his3* Δ *1 leu2-3,112/leu2* Δ *0 ura3-1 trp1-1 ade2* Δ *::kanMX pDal5::ADE2 ure2* Δ *::cyh2 lys2* Δ *::pGPD-CD-FD*	This study
Prion CD-FD 2n (1856)	*MAT***a***/* α *his3* Δ *1 leu2-3,112/leu2* Δ *0 ura3-1 trp1-1 ade2* Δ *::kanMX pDal5::ADE2 ure2* Δ *::cyh2 lys2* Δ *::pGPD-CD-FD* (Ade ${}^{+}$ White)	This study
Prion CD-FD (1870)	*MAT* α *his3* Δ *1 leu2* Δ *0; ura3-1 trp1-1 ade2* Δ *::kanMX pDal5::ADE2 URE2::cyh2 lys2* Δ *::pGPD-CD-FD* (Ade ${}^{+}$ White)	This study
Prion CD-FD (1869)	*MAT* α *his3* Δ *1 leu2* Δ *0 ura3-1 trp1-1 ade2* Δ *::kanMX pDal5::ADE2 ure2* Δ *::cyh2 lys2* Δ *::pGPD-CD-FD* (Ade ${}^{+}$ Pink)	This study
Prion CD-FD (1871)	*MAT***a***his3* Δ *1 leu2-3,112 ura3-1 trp1-1 ade2* Δ *::kanMX pDal5::ADE2 ure2* Δ *::cyh2 lys2* Δ *::pGPD-CD-FD* (Ade ${}^{+}$ White)	This study
Prion CD-FD (1868)	*MAT***a***his3* Δ *1 leu2-3,112 ura3-1 trp1-1 ade2* Δ *::kanMX pDal5::ADE2 ure2* Δ *::cyh2 lys2* Δ *::pGPD-CD-FD* (Ade ${}^{+}$ Pink)	This study
GPD-URE2 (2037)	*MAT***a***his3* Δ *1 leu2-3,112 ura3-1 trp1-1 ade2* Δ *::kanMX pDal5::ADE2 ure2* Δ *::cyh2 lys2* Δ *::pGPD-Ure2FL*	This study
Prion [URE3] (1207)	*MAT***a***trp1-1 ade2-1 leu2-3,112 his3-11,15 ura2* Δ *::HIS3 pdal5::ADE2* [*URE3*]	23
SB 34 Prion [URE3] (287)	*MAT* α *trp1-1 ade2-1 leu2-3 112 his3-11,15 ura2* Δ *::HIS3 pdal5::ADE2* [*URE3*]	23
CD-FD cytoduction recipient (2112)	*MAT***a***kar1 trp1 his3 leu2 ure2* Δ *ade2 pDal5::ADE2 lys2* Δ *::pGPD-CD-FD* [*rho* ${}^{\circ }$ ]	This study
L3671	*MAT* α *ade2-1 leu2-3,112 trp1-1 his3-11,15 can1-100 URA3::3xRGC::p-cyc1::ADE2::ura3-1* transformed with pADH1-p53 (*LEU2*) and pGAL1-p53-EYFP (*TRP1*)	14
L3672	L3671 with p53 prion	14
L3664	*MAT***a***kar1 ura2 leu2 his3* [*rho* ${}^{\circ }$ ]	14

### Crispr approach

p414-TEF1p-Cas9-CYC1t (Addgene) allows expression of Cas9 from the *TEF1* promoter. The RNA guide (gRNA) is produced from p426-SNR52p-gRNA. We changed the selectable marker of this plasmid by co-transformation with a PCR product in which *HIS3* is flanked by UTR sequences of *URA3*. This amplification was done with oligonucleotides 1195 and 1196, the matrix being pGal-ccdB (809). Finally, hybridization of oligonucleotides 1197 and 1198 or 1199 and 2000 led to overhang extremities that can be inserted in this plasmid after AarI digestion. The P426gRNA1-Lys2 (755) and P426gRNA2-Lys2 (756) contain both gRNA for *LYS2* targeting two different loci.

### ure2 Δ strains

BY4742 Δ *ade2* (*MAT* α *his3* Δ *1 leu2* Δ *0 lys2* Δ *0 ura3* Δ *0 ade2* Δ *::kanMX*) was mated to NT62 (*MAT***a***trp1-1 ade2-1 his3-11,15 leu2-3,112 ura3-1 pdal5::ADE2 ure2* Δ *::cyh2*), and diploids were selected as Kan ${}^{R}$ , Lys ${}^{+}$ . After meiosis, spores were selected for the phenotypes Kan ${}^{R}$ , Ade ${}^{+}$ , Lys ${}^{+}$ , Trp ${}^{-}$ , His ${}^{-}$ , Leu ${}^{-}$ . The corresponding genotype is (*ade2* Δ *::kanMX pDal5::ADE2 ure2* Δ *::cyh2 trp1-1 his3* Δ *1 leu2-3,112 ura3-1*) and correspond to the MK1 *MAT***a** and MK2 *MAT* α strains.

### CD-FD strains

The *MAT***a** CD-FD strain was obtained by integrating the expression cassette of pGPD-CD-FD at the *LYS2* locus in the MK1 strain by a CRISPR-Cas9 strategy 56. The MK1 strain was transformed by p414-TEF1p-Cas9-CYC1t (Addgene). Trp ${}^{+}$ clones were grown to mid-log phase and then transformed with gRNA-LYS2 vectors that generate a guide RNA (gRNA) for the Cas9 nuclease that is specific for the *LYS2* locus. The double strand break at the *LYS2* locus is repaired by a PCR product obtained with the oligonucleotides 1189 and 1190 and the matrix pGPDH-CD-FD (810). The resulting cassette consists of a CD-FDp expression cassette flanked by *LYS2*-homologous sequences (*LYS2*-GPD-CD-FD-*LYS2*). His ${}^{+}$ , Trp ${}^{+}$ clones were selected on semi-complete medium lacking tryptophane and histidine and checked for lysine auxotrophy (they should become Lys ${}^{-}$ ).

The *MAT* α CD-FD strain was obtained by backcrossing the *MAT***a** CD-FD strain with the MK2 strain. Zygotes were manually isolated with the Singer apparatus. After meiosis, spores were selected as (*MAT* α *his3* Δ *1 leu2* Δ *0 ura3-1 trp1-1 ade2* Δ *::kanMX pDal5::ADE2 ure2* Δ *::cyh2 lys2* Δ *::pGPD-CD-FD*).

For cytoduction, the *kar1* allele was introduced by crossing the CD-FD *MAT* α strain (*MAT* α *his3* Δ *1 leu2* Δ *0 ura3-1 trp1-1 ade2* Δ *::kanMX pDal5::ADE2 ure2* Δ *::cyh2 lys2* Δ *::pGPD-CD-FD*) with strain BY188 (*MAT***a***ade2 his3 leu2-3,112 ura2* Δ *ure2* Δ *pDal5::KanMx pDal5::CAN1 kar1*) 55. Diploids were selected on synthetic medium without uracil. After meiosis, several Ura ${}^{+}$ , Ade ${}^{-}$ on nitrogen rich medium and Ade ${}^{+}$ in nitrogen poor medium, Lys ${}^{-}$ spores were isolated. Among these spores, half possess the *kar1* mutation (*kar1* mutation causes a weak, delayed response in standard complementation test and can be easily tested in a mating test). Finally, a (*MAT***a***ure2* Δ *pDal5::ADE2 lys2* Δ *::pGPD-CD-FD kar1*) strain was selected and streaked on YPD in the presence of ethidium bromide (25 μ g/mL). After 4 days at 30 ${}^{\circ }$ C, independent clones were tested on complete medium and YPgly respiratory medium. A respiratory-deficient clone [*rho* ${}^{\circ }$ ] was selected as a cytoduction recipient strain.

### CD-FD prion strains

Yeast cells were cultivated overnight in plasmid-selective synthetic medium (pGPDW-CD-FD MK1 transformants) or in complete medium (CD-FD 2n). Around 10 ${}^{7}$ cells were spread on synthetic medium lacking adenine. Several clones were tested for the presence of CD-FDp by western blot. Three Ade ${}^{+}$ diploids expressing the chimeric protein were sporulated and dissected on YPD. Plates were incubated at 30 ${}^{\circ }$ C for 2 to 3 days. From two 2n Ade ${}^{+}$ clones, we could observe several tetrads with three white colonies and one red colony. They were tested on a medium without adenine to confirm the three Ade ${}^{+}$ / one Ade ${}^{-}$ segregation. Prion strains 1870 and 1871 were selected from one tetrad: they are white and grow steadily on media lacking adenine. Prion strains 1868 and 1869 were selected from a second tetrad: they are pink and grow more slowly on adenine.

Disruption of *HSP104* was done as described in 40 using the plasmid pYABL5 (kindly provided by Dr Susan Lindquist). Briefly, the 4.3 kb PvuII-HindIII fragment containing the *LEU2* gene was used to transform the strain 1870. The Leu ${}^{+}$ transformants were tested for reduced viability after prolonged incubation at 5O ${}^{\circ }$ C.

### Protein expression analysis

Total native yeast protein extracts were prepared by glass bead disruption. A culture (50 mL) in mid-log phase (DO ${}_{\mathrm{650nm}}$ ∼ 2) was centrifugated for 5 min at 3,000 g. The pellet was resuspended in 500 μ L TNTG (10 mM Tris pH 7.4, 5 mM EDTA, NaCl 0.2 M, 0.1% Triton 100 X, 10% glycerol) containing 1 mM phenylmethylsulfonyl fluoride. 300 μ L of glass microbeads (0.5 mm) were added before vigorous agitation using a MP Biomedicals FASTPREP®-24 device for 30 s at speed 6 m/s followed by a centrifugation for 10 min (5,000 g, 4 ${}^{\circ }$ C). The supernatant was collected, yielding the total protein extract (T), which was then fractionated by centrifugation at 100,000 g for 60 min. The resulting supernatant was recovered and replaced with an equal volume of TNTG to generate the pellet fraction. Identical volumes of each fraction were incubated for 10 min at 100 ${}^{\circ }$ C in 1X loading buffer (0.06 M Tris-Cl, pH 6.8, 2% SDS, 10% glycerol, 5% β -mercaptoethanol, 0.002% bromophenol blue) and separated by 12% SDS-PAGE. Proteins were electrically transferred onto nitrocellulose membranes (Optitran BA-S83, Schleicher & Schuell) in the presence of transfer buffer (39 mM glycine, 48 mM Tris-base, 2% ethanol and 0.037% SDS) and were probed with monoclonal DO-12 mouse anti-p53 antibodies at 1 μ g/mL (kindly provided by R.Iggo). Peroxidase-conjugated anti-mouse antibodies (Sigma) were used as secondary antibodies. Binding was detected with the SuperSignal reagent (Thermo) and the ChemiDoc Imaging system (BioRad). For actin detection, the membrane was soaked in a stripping solution (0.1 M glycine, 1% SDS, 1% Tween 20) and reprobed with anti-actin antibodies at 1 μ g/mL (kindly given by F. Doignon).

An alternative non-native alkaline lysis extraction method was used. Briefly, 5 OD ( λ =600 nm) units of yeast cells in exponential growth were permeabilized with 500 μ L of 0.185 M NaOH, 0.2% β -mercaptoethanol. After a 10 min incubation on ice, trichloroacetic acid (TCA) was added to a final concentration of 5%, and the samples were incubated for an additional 10 min on ice. Precipitates were then collected by centrifugation at 21,000 g and solubilized in 50 μ L of resuspension buffer (1X loading buffer + Tris base 50 mM). 2 to 5 μ L were analyzed by SDS-PAGE.

### Fluorescence microscopy and Thioflavin T staining

To investigate the diffuse or aggregated state of p53, CD-FD prion (1868, 1869, 1870 and 1871) and non-prion (1844, 1851 and 1855) strains were transformed with pAG413 pGPD-p53-EYFP (*CEN, HIS3*). Transformants were selected and patched on plasmid selective medium lacking histidine. Cells were imaged after 2 days of incubation at 30 ${}^{\circ }$ C. EYFP fluorescence imaging was done on six independent transformants for each strain, with 80 to 400 cells examined for each transformant.

To assess whether p53-EYFP foci colocalized with Th-T positive aggregates, non-prion (1844) and prion (1869 and 1871) CD-FD strains as well as subclones of non-prion (L3671) and prion (L3672) FLp53 strains were transformed with pAG413 pGPD-p53-EYFP (*CEN, HIS3*). Transformants were selected on dextrose-based plasmid-selective medium lacking histidine and grown for 2 days on SD–His plates to allow expression of p53-EYFP. Cells were visualized after being fixed as described 57 and stained with Thioflavin T. EYFP and Th-T fluorescence imaging was done on three independent clones for each strain, with 150 to 800 cells examined for each clone.

For cytoplasm transfer experiment, selected cytoductants were grown 2 days at 30 ${}^{\circ }$ C on plasmid selective medium lacking histidine then imaged for EYFP fluorescence. Imaging was done on 150 to 400 cells for six independent non-prion and ten independent prion cytoductants.

Imaging was done with a Nikon Eclipse E600 fluorescent microscope (60X oil immersion) equipped with YFP (for EYFP fluorescence) and CFP (for ThT staining) filter cubes.

### Cytoduction

Prion strains CD-FD (1869) (*MAT* α *his3* Δ *1 leu2* Δ *0 ura3-1 trp1-1 ade2* Δ *::kanMX pDal5::ADE2 ure2* Δ *::cyh2 lys2* Δ *::pGPD-CD-FD*) and SB34 [*URE3*] (*MAT* α *trp1-1 ade2-1 leu2-3,112 his3-11,15 ura2* Δ *::HIS3 pdal5::ADE2)* were grown for 2 days on complete respiratory medium YPGly while CD-FD recipient strain (2112) (*MAT***a***, kar1 trp1 his3 leu2 ure2* Δ *ade2 pDal5::ADE2 lys2* Δ *::pGPD-CD-FD* [*rho* ${}^{\circ }$ ]) was grown on complete medium. Donor and recipient strains were then mated for 7 h on complete medium. The mating cells were resuspended in water and around 10 ${}^{4}$ cells spread on respiratory N4W medium lacking uracil and complemented or not with adenine. Plates were incubated for a week at 30 ${}^{\circ }$ C.

The recipient strain L3664 (*MAT***a***kar1 ura2 leu2 his3* [*rho* ${}^{\circ }$ ]) was transformed with pGPD-p53-EYFP (*CEN, HIS3*) and selected on plasmid selective medium lacking histidine. Transformants were mated on complete medium with *MAT* α CD-FD prion (1869, 1870) and non-prion strains (1844) (*MAT* α *his3* Δ *1 leu2* Δ *0 ura3-1 trp1-1 ade2* Δ *::kanMX pDal5::ADE2 ure2* Δ *::cyh2 lys2* Δ *::pGPD-CD-FD*) until zygotes were observed (around 4–6 h). The mating mixture was then streaked on plasmid selective medium lacking histidine SD-His which selects for cytoductants, recipients, and diploids. His ${}^{+}$ clones were then replicated on non-fermentable medium YPGly containing 2% glycerol instead of glucose. [*RHO* ${}^{+}$ ] cells (which eliminates recipients) that were confirmed to be (His ${}^{+}$ , Trp ${}^{+}$ , Ura ${}^{-}$ , Leu ${}^{-}$ ) were picked from SD-His and patched for fluorescence imaging.

### Fluctuation assay

Transformants or integrants were grown overnight in an adenine-complemented medium either plasmid-selective or complete respectively. The culture was diluted at 1% in the same medium in order to reduce probability of getting cells in which CD-FDp is already inactivated. 200 μ L of the dilution were grown into 16 micro wells for 24 h at 30 ${}^{\circ }$ C. Absorbance was measured in order to estimate the number of cells per wells, and all cultures (48 for three independent clones) were spread on synthetic medium lacking adenine (and plasmid-selective for transformants). Plates were incubated for 3 days and Ade ${}^{+}$ colonies were counted. The number of Ade ${}^{+}$ clones were treated by an algorithm based on maximum likelihood (available online at https://websalvador.eeeeeric.com/ 58) in order to extract inactivation frequency per generation.

## DATA AVAILABILITY

Strains and plasmids are available upon request. The authors affirm that all data necessary for confirming the conclusions of the article are present within the article, figures, and tables.

## SUPPLEMENTAL MATERIAL

All supplemental data for this article are available online at http://www.microbialcell.com/researcharticles/2026a-kadouch-microbial-cell/. 

## CONFLICT OF INTEREST

The authors declare no competing interests. The authors received no financial support for the research, authorship, and/or publication of this article.
