# Supplementary material for: The p53 DNA-Binding (Core) Domain can form a prion that seeds prion formation in full-length p53
Source: Microb Cell. 2026 Oct 2;13:340–57. doi: 10.15698/mic2026.10.887 (PMC13646917; doi:10.15698/mic2026.10.887)
Supplement: Supplementary file 1 [file mic-13-340-s01.pdf]

The p53 DNA-Binding (Core) Domain can form a prion that seeds prion formation in full-length p53

SUPPLEMENTAL INFORMATION

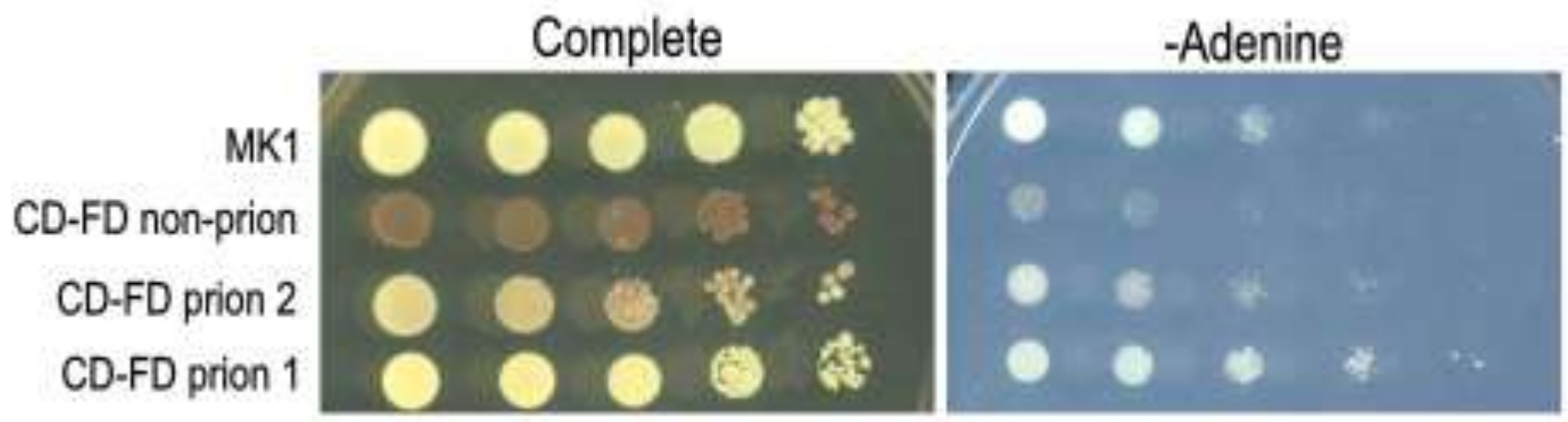

Figure S1. MK1 (1840), CD-FD non prion (1844), CD-FD prion 1 (1870) and 2 (1869) strains were streaked on complete medium. After 3 days of incubation, one isolated colony of each strain was serially diluted and spotted and complete medium and on synthetic medium lacking adenine. Cells were observed after 3 days of incubation.

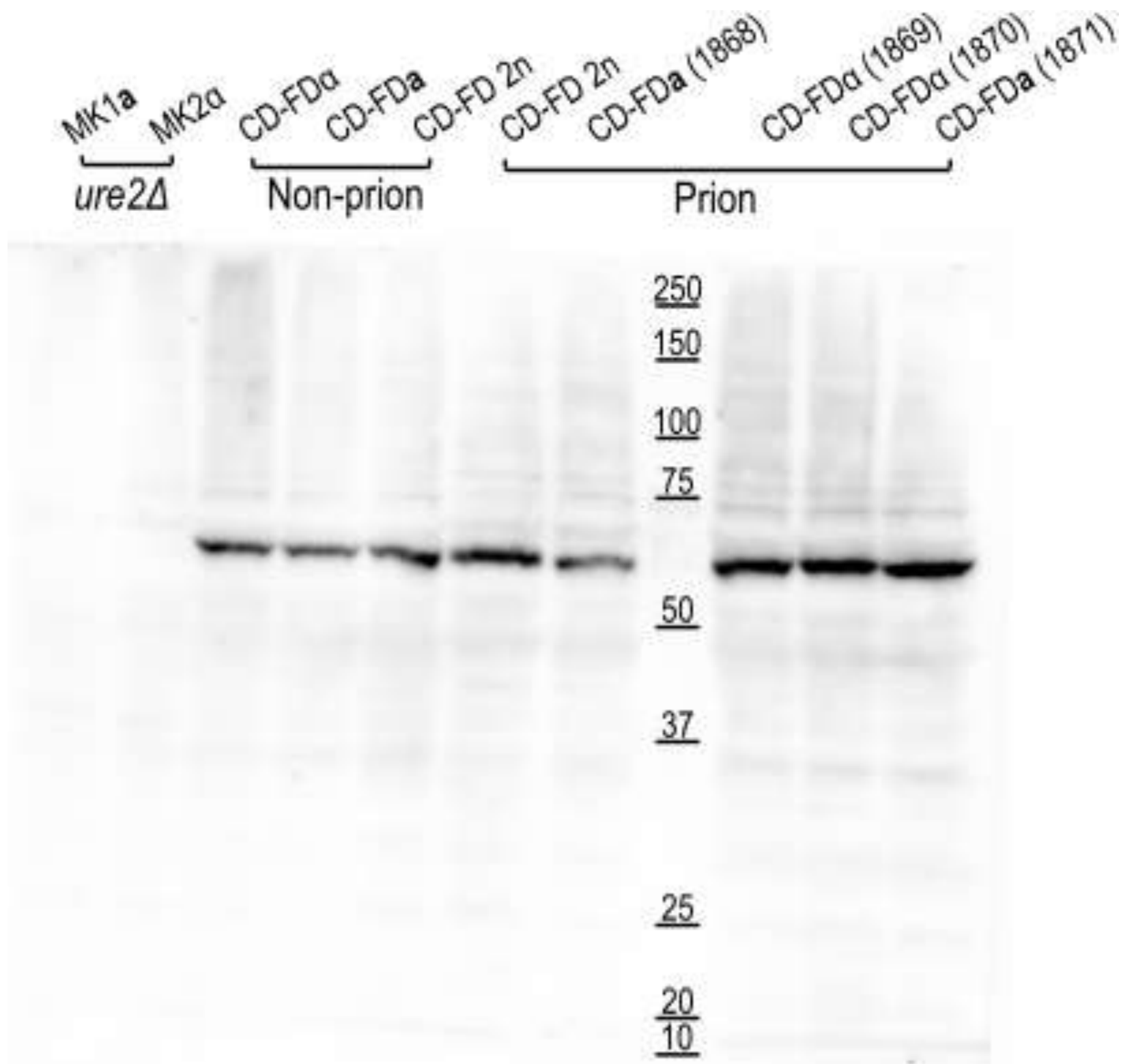

Figure S2. CD-FDp expression in prion and non-prion strain. Strains were grown on complete medium and raw total protein extracts were prepared as described in the methods section. Protein extracts were then separated by SDS-PAGE, transferred to a nitrocellulose membrane, and probed for CD-FDp using DO-12 anti-p53 antibodies. Underlined are indicated molecular weights.

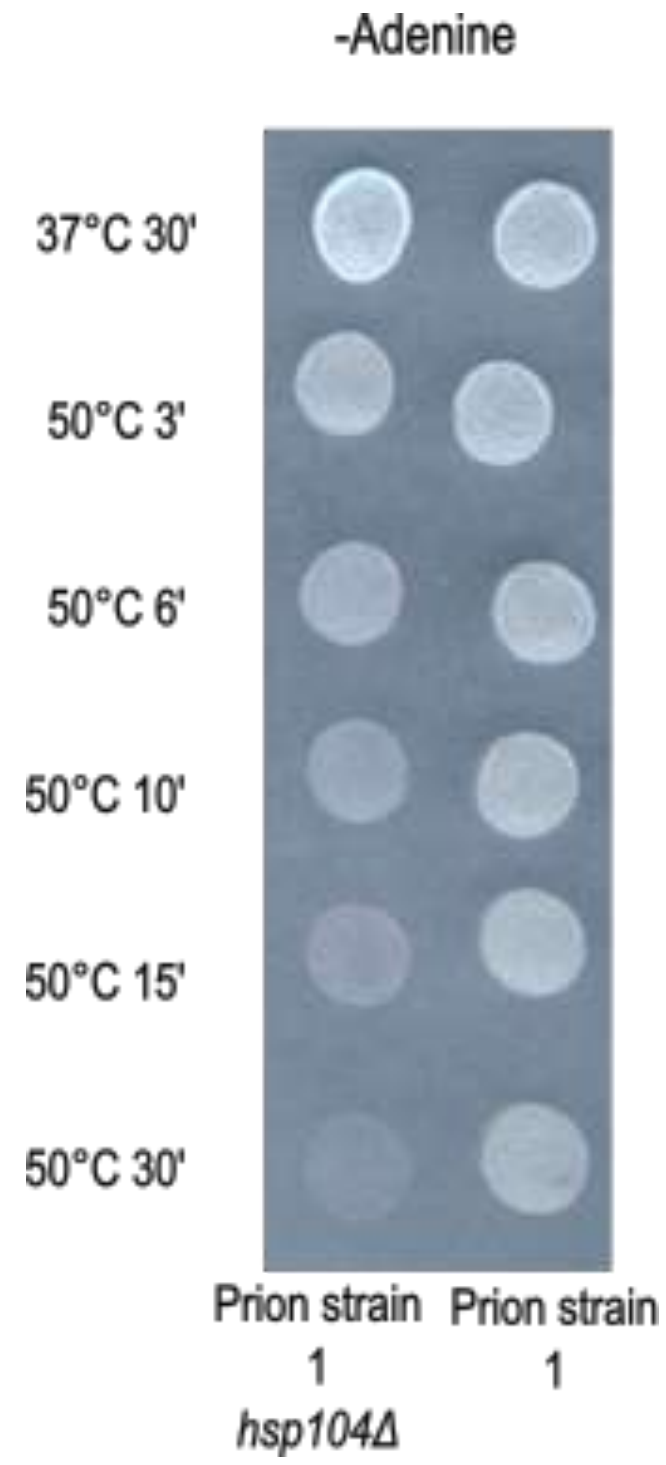

Figure S3. *HSP104* deletion does not cure CD-FDp prion. *HSP104* was disrupted in prion strain 1 (1870) by replacing the *HSP104* gene with a *HSP104::LEU2* cassette. An Ade+, Leu+ clone was then tested for thermotolerance. Yeast cells were pre-incubated for 30 minutes at 37°C, followed by exposure to 50°C for varying durations. After each time point, aliquots were rapidly chilled on ice before plating and incubated at 30°C for 24 hours on synthetic medium lacking adenine.

Table S1. CD-FDp inactivation frequency (plasmidic expression)

pGPD-CD-FD		Ade+ clones																OD	Nt	Mean 1	Mean 2
Exp. 1	Clone 1	4	143	40	1	6	5	30	65	37	116	116	72	2	28	3	5	5,6	13440000		
	Clone 2	34	34	193	134	14	26	33	30	162	38	15	19	10	61	294	7	5,1	12240000	13040000	
	Clone 3	48	24	111	298	24	12	41	75	1	5	9	5	2	1	3	0	5,6	13440000		14880000
Exp. 2	Clone 4	12	5	7	1	7	7	3	4	20	34	4	2	36	105	37	15	8,6	20640000		
	Clone 5	0	4	3	1	6	20	49	18	10	21	4	8					6,8	16320000	16720000	
	Clone 6	0	0	0	0	1	1	6	1	28	4	1	0					5,5	13200000		

Shown are the number of Ade+ colonies derived from each microwell. In two experiments, six independent transformants were grown until saturation in synthetic plasmid-selective medium. The cultures were diluted at 1/100 and 200 µl of the dilution was grown into 16 microwells until saturation. Absorbance was measured and the cultures were spread on semi-complete plasmid-selective medium lacking adenine. Ade+ clones were counted after 3 days at 30°C. OD – absorbance; Nt – Estimated number of cells per culture; Mean – mean number of cells per culture.

Table S2. CD-FDp inactivation frequency – Clone to clone variability

	**Individual measures**			**pvalue**				
	lower	point est	upper	C1 vs Cx	C2 vs Cx	C3 vs Cx	C4 vs Cx	C5 vs Cx
C1	2,03E-07	3,15E-07	4,54E-07					
C2	5,25E-07	7,33E-07	9,66E-07	**8.7789e-04**				
C3	1,44E-07	2,32E-07	3,44E-07	3.0813e-01	**1.5918e-05**			
C4	1,14E-07	1,73E-07	2,46E-07	**3.7497e-02**	**3.9948e-08**	3.2720e-01		
C5	1,02E-07	1,75E-07	2,67E-07	7.0822e-02	**4.3333e-07**	4.2544e-01	9.2189e-01	
C6	2,57E-08	5,91E-08	1,12E-07	**1.5339e-05**	**5.7110e-13**	**7.0486e-04**	**5.2596e-03**	**9.5200e-03**

Individual measures were compared using Mandelbrot-Koch model. Bolded pvalues indicate that the clones compared gave significantly different inactivation frequencies.

Table S3. Variant CD-FDp inactivation frequency (plasmidic expression)

pGPD-CD*-FD		Ade$^+$ clones																OD	Nt	Mean
248W 142T	Exp. 1	52	73	167	189	99	86	65	75	138	126	94	55	125	192	103	77	7,8	18720000	14742857,1
		5	12	1	150	114	33	97	15	4	4	3	0	25	5	19	12	6,2	14880000	
		3	15	165	31	7	82	63	43	23	73	36	18	107	36	46	93	6,2	14880000	
	Exp. 2	0	1	6	19	80	6	0	0	15	23	26	127	21	30	6	111	5	12000000	
		22	22	2	10	9	15	34	55	54	12	11	31	150	11	19	122	4,9	11760000	
		3	6	9	11	0	5	54	150	35	8	12	37	28	3	44	6	5,9	14160000	
	Exp. 3	0	5	6	53	0	4	8	14	1	3	3	10	2	1	9	9	7	16800000	
		6	56	1	8	6	3	17	27	6	3	2	5	81	12	2	5	7,8	18720000	
		133	150	150	107	150	150	141	150	150	150	49	90	150	150	150	150	5,5	13200000	
273H	Exp. 1	17	0	0	5	138	15	5	13	150	19	8	55	13	15	3	0	6,7	16080000	15888000
		46	150	150	150	133	38	33	69	7	13	11	12	42	87	150	150	7,8	18720000	
		3	4	0	0	31	4	11	58	0	1	1	0	11	13	14	7	6,1	14640000	
	Exp. 2	2	2	4	114	2	3	36	22	48	127	81	3	10	4	77	79	6,2	14880000	
		36	114	29	9	0	1	7	24	14	106	150	7	6	6	2	22	6,3	15120000	
		150	150	150	150	150	150	150	150	150	150	150	150	150	150	150	150	6,7	16080000	
249S	Exp. 1	9	6	6	5	28	87	23	52	9	18	68	24	2	3	7	2	4,6	11040000	12200000
		35	66	6	25	8	71	78	28	71	24	43	151	7	62	18	155	3,9	9360000	
		24	3	3	4	7	1	0	0	15	1	1	2	1	3	27	12	4,5	10800000	
	Exp. 2	30	17	17	24	67	24	45	20	24	2	3	2	29	11	11	6	5,9	14160000	
		6	16	23	4	12	14	25	15	40	3	8	9	107	31	15	6	5,9	14160000	
		86	53	45	71	2	19	44	144	14	11	74	54	98	91	147	38	5,7	13680000	
282W	Exp. 1	4	10	75	2	1	9	71	59	150	147	94	48	1	9	108	19	6,4	15360000	12160000
		0	0	7	101	0	0	1	6	7	5	57	24	2	12	33	0	5,2	12480000	
		8	7	5	24	0	0	0	0	2	2		3	6	7	3	1	5,9	14160000	
	Exp. 2	4	10	73	2	1	9	94	48	1	5	108	15	71	59	150	147	3,2	7680000	
		0	0	7	101	0	0	0	1	6	7	5	57	24	2	12	33	4,2	10080000	
		8	7	5	24	0	0	0	0	2	2	3	12	6	7	3	1	5,5	13200000	

Shown are the number of Ade$^+$ colonies derived from each microwell. In 2 or 3 experiments, 6 to 9 independent transformants were grown until saturation in semi-complete plasmid-selective medium. The cultures were diluted at 1/100 and 200 µl of the dilution was grown into 16 microwells until saturation. Absorbance was measured and the cultures were spread on semi-complete plasmid-selective medium lacking adenine. Ade$^+$ clones were counted after 3 days at 30°C. OD – absorbance; Nt – Estimated number of cells per culture; Mean – mean number of cells per culture. Colored in orange are "lucky winner" that already contained Ade$^+$ cells in the dilution. They were not used for inactivation frequency estimations.

Table S4. CD-FDp and CD^{R175H}-FDp inactivation frequency (genomic expression)

	Ade$^+$ clones																OD	Nt	Mean	Spread fraction
	10	12	8	21	3	42	7	28	150	136	150	150	22	88	22	150	8,4	20160000		
**WT**	4	3	0	0	1	1	0	4	3	2	3	0	0	0	0	0	9,3	22320000	21040000	1
	6	6	1	4	3	1	0	0	111	121	81	124	0	0	0	0	8,6	20640000		
	65	40	55	144	150	75	72	145	113	111	26	150	45	2	5	150	4,1	9840000		
**R175H**	150	150	150	150	150	150	150	150	150	150	150	150	150	150	150	150	5,2	12480000	11880000	0.1
	5	4	5	4	1	3	0	0	9	3	3	8	4	8	14	8	5,8	13920000		

Shown are the number of Ade$^+$ colonies derived from each microwell. Three independent transformants were grown until saturation in complete medium. The cultures were diluted at 1/100 and 200 µl of the dilution was grown into 16 microwells until saturation. Absorbance was measured and the cultures were spread on semi-complete medium lacking adenine. The CD-FD strain (WT, 1851) culture was totally spread while only one-tenth of the CD^{R175H}-FD strain culture (R175H) was spread. Ade$^+$ clones were counted after 3 days at 30°C. OD – absorbance; Nt – Estimated number of cells per culture; Mean – mean number of cells per culture. Colored in orange are “lucky winner” that already contained Ade$^+$ cells in the dilution. They were not used for inactivation frequency estimations.
